# Exploring the Transitivity Assumption in Network Meta‐Analysis: A Novel Approach and Its Implications

**DOI:** 10.1002/sim.70068

**Published:** 2025-04-10

**Authors:** Loukia M. Spineli, Katerina Papadimitropoulou, Chrysostomos Kalyvas

**Affiliations:** ^1^ Midwifery Research and Education Unit Hannover Medical School Hannover Germany; ^2^ Health Economics and Market Access, Amaris Consulting Lyon France; ^3^ Biostatistics and Medical Informatics, Medical Faculty University of Ljubljana Ljubljana Slovenia

**Keywords:** dendrogram, dissimilarity matrix, heatmap, network meta‐analysis, transitivity

## Abstract

The feasibility of network meta‐analysis depends on several factors, one of which is the validity of the transitivity assumption that posits no systematic differences in the distribution of effect modifiers across treatment comparisons within a connected network. However, evaluating transitivity is complex for relying on epidemiological grounds. Therefore, establishing a methodological framework to evaluate this assumption is challenging. We propose a novel approach, which involves calculating dissimilarities between treatment comparisons based on study‐level aggregate participant and methodological characteristics reported across studies and applying hierarchical clustering to cluster similar comparisons. This approach detects “hot spots” of potential intransitivity in the network, enabling empirical exploration of transitivity and semi‐objective judgments. Our approach quantifies clinical and methodological (non‐statistical) heterogeneity within and between treatment comparisons by computing the dissimilarities across studies in key characteristics acting as effect modifiers. The investigated networks showed varying between‐comparison dissimilarities, indicating variability in the clinical and methodological heterogeneity of the networks. Several pairs of treatment comparisons with “likely concerning” non‐statistical heterogeneity were identified, and some studies were organized into several clusters, suggesting potential intransitivity in the networks. These findings necessitate a closer examination of the evidence base, and such scrutiny becomes pivotal in determining whether concerns about the feasibility of network meta‐analysis are justified. Similar to statistical heterogeneity, heterogeneity in clinical and methodological characteristics of the collected studies should be expected and appropriately assessed. Our proposed approach facilitates the evaluation of transitivity using well‐established methods and can be applied to newly planned and published systematic reviews.

## 1 Introduction

Systematic reviews involving multiple treatments have been at the forefront of evidence synthesis research due to the wealth of information they provide to end users. Over the years, extensive research efforts have gradually contributed to the development and widespread dissemination of network meta‐analysis (NMA), the extension of pairwise meta‐analysis for multiple treatments [1]. Researchers have dedicated substantial efforts to creating and assessing various statistical methods, understanding NMA assumptions, improving reporting practices, and exploring approaches to assess confidence in the NMA results [2, 3, 4, 5, 6].

The credibility and reliability of NMA results depend, among other factors, on the validity of the transitivity assumption. Transitivity posits that there should be no systematic differences in the distribution of the effect modifiers across treatment comparisons within a connected network [3, 6]. In other words, the studies informing the treatment comparisons should not differ beyond the treatments being compared [3, 7]. Then, it can be inferred that participants comprising the target population of the systematic review could be randomized to any treatment within the network, and treatments not being investigated in a study may be missing for reasons unrelated to their actual effects [3, 8]. Essentially, transitivity ensures the feasibility and validity of performing NMA. However, evaluating transitivity is challenging due to the epidemiological nature of this assumption, necessitating a deep understanding of the disease area, treatment landscape, and relevant effect modifiers [9, 10]. It also requires access to comprehensive study reports to make informed judgments.

Current practices for assessing transitivity involve graphical and statistical explorations of study‐level aggregate participant and methodological characteristics acting as effect modifiers. Analysts visualize the distribution of each characteristic across comparisons using graphs, such as bar plots or box plots, to gauge the similarity of comparisons. A network plot can also be employed by adjusting the edge thickness to reflect the relative frequency or average value of a characteristic of interest (e.g., the proportion of females or the average age) in the comparisons [8]. Alternatively, pie charts or histograms on the network's edges can illustrate the distribution of a characteristic [11]. Statistical tests, such as the chi‐squared test or one‐way ANOVA, can also be performed to assess the comparability of comparisons for each characteristic if sufficient studies inform the comparisons and characteristics are well reported.

However, graphical evaluation relies heavily on subjective judgments and is conditional upon (a) the number of studies informing the comparisons and (b) the availability of the investigated characteristics. While statistical evaluation is straightforward and objective, it can be susceptible to multiplicity when applied to each characteristic individually. One approach to mitigate the multiplicity issue is to simultaneously test the comparability of comparisons for all characteristics or adjust the significance level. Nevertheless, in networks where comparisons are sparsely informed and many characteristics are missing, statistical testing will be unreliable as it may lack the power to detect a statistically significant intransitivity, even if it exists for only one characteristic.

Alternatively, dissimilarity measures and unsupervised learning methods, such as clustering, could be used to gauge the dissimilarity between treatment comparisons and identify clusters based on important effect modifiers. Clustering will group highly similar treatment comparisons together while separating dissimilar ones into different clusters. While clustering has limitations, such as dealing with sparse comparisons and missing characteristics, its unsupervised nature does not rely on hypothesis testing. Instead, it relies on well‐established dissimilarity measures and algorithms that offer an *exploratory* analysis, revealing potential patterns in treatment comparisons [12]. Additionally, the quality of clustering can be assessed using objective validity measures supplemented by subjective judgments, if needed. Thus, clustering and its building block, dissimilarity measures, could allow semi‐objective inferences regarding the plausibility of transitivity, even in parts of the network with more available information.

Hierarchical clustering has previously been proposed in the context of NMA to identify clusters of treatments that rank similarly based on two outcomes [13]. This valuable tool has been applied in many published systematic reviews involving multiple treatments. Introducing dissimilarity measures and clustering into the transitivity evaluation framework presents a novel approach to thoroughly examine the comparability of treatment comparisons, offering a rich visualization toolkit and established methods. Ultimately, dissimilarity measures and clustering can help identify potential areas of intransitivity within the investigated network of treatments, prompting further scrutiny of the evidence base to determine whether concerns about the feasibility of NMA are warranted.

The article is structured as follows: Section 2 presents two published systematic reviews, each with a different number of treatments and network structures. Section 3 introduces the framework for transitivity evaluation, providing a step‐by‐step explanation of the methodology. In Section 4, we apply our framework to the motivating examples. Section 5 elaborates on the results, highlights the advantages and limitations of the framework, and proposes a research agenda for further work on transitivity evaluation. Section 6 offers recommendations for using and benefiting from the framework in a systematic review with multiple treatments.

## 2 Motivating Examples

**FIGURE 1:**
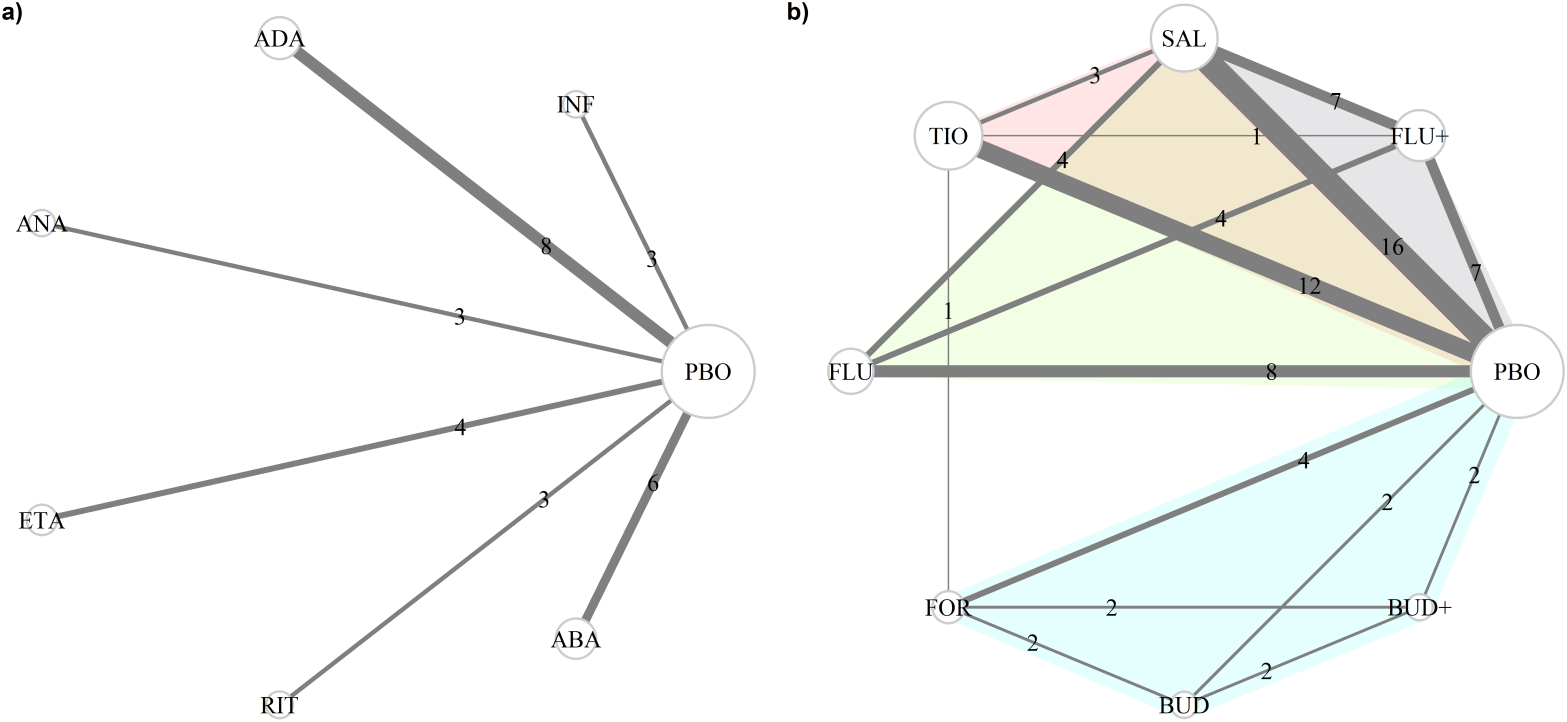
Network on six biologic disease‐modifying anti‐rheumatic agents and placebo (plot a) [14]. Network on seven pharmacologic treatments and placebo for chronic obstructive pulmonary disease (plot b) [15]. The size of the nodes is proportional to the total number of patients receiving the corresponding treatment, and the thickness of the edges is proportional to the number of studies investigating the corresponding comparison. ABA, abatacept; ADA, adalimumab; ANA, anakinra; BUD, budesonide; BUD+, budesonide plus formoterol; ETA, etanercept; FLU, fluticasone; FLU+, fluticasone plus salmeterol; FOR, formoterol; INF, infliximab; PBO, placebo; RIT, rituximab; SAL, salmeterol; TIO, tiotropium.

In our first example, we consider the Cochrane review by Singh et al. [14], focusing on six biologic disease‐modifying anti‐rheumatic agents: abatacept (ABA), adalimumab (ADA), anakinra (ANA), etanercept (ETA), infliximab (INF), and rituximab (RIT) as well as placebo (PBO). The primary outcome is a 50% improvement from baseline to endpoint, addressing “the number of tender or swollen joints and other doctor or patient assessed aspects of rheumatoid arthritis” [14]. All active agents were compared against PBO, yielding a star‐shaped network (Figure 1a). The analysis dataset includes 27 studies (Supporting Information S1, Table S1) and 10 *study‐level aggregate* characteristics, which could be extracted for all studies, including:

- three quantitative characteristics: total sample size, study duration (in months), and disease duration (in years), and
- seven qualitative characteristics: concomitant use of methotrexate (MTX), rheumatoid arthritis duration (early, established, or late), whether the biologic is anti‐tumor necrosis factor (TNF), prior drugs failed, prior failure of TNF biologic, combination of biologic therapy, and naïve biologic.

The second example is a systematic review conducted by Baker et al. [15], investigating seven pharmacologic treatments (budesonide [BUD], budesonide plus formoterol [BUD+], fluticasone [FLU], fluticasone plus salmeterol [FLU+], formoterol [FOR], salmeterol [SAL], tiotropium [TIO]) and PBO for the primary outcome of exacerbation of chronic obstructive pulmonary disease (COPD) episodes (Figure 1b). The dataset comprises 39 studies and 11 *study‐level aggregate* characteristics (Supporting Information S1, Table S2), including:

- eight quantitative characteristics: total sample size, study duration (in weeks), percentage forced expiratory volume in 1 s (FEV_1_) at inclusion, percentage forced vital capacity (FVC) at inclusion, smoking history (cigarette packets per year), aggregate quality score, the minimum and maximum mean percentage of FEV_1_, and
- three qualitative characteristics: random allocation concealment, double blinding, and withdrawal description. We did not consider the definition of COPD exacerbation, as it required an expert opinion for the categorization (table 1 in [15]).

These two networks present varying degrees of difficulty in determining the optimal number of clusters. They serve as ideal examples for illustrating the advantages and limitations of the proposed framework for transitivity evaluation and discussing viable solutions for addressing the challenges encountered.

## 3 Methods

### 3.1 Gower's Dissimilarity Coefficient Across Studies

We need a suitable metric to calculate the dissimilarity between two studies across several study‐level aggregate characteristics, which serve as effect modifiers. Since these characteristics may include a mix of quantitative and qualitative data (i.e., binary, nominal, and ordinal variables), we employ Gower's dissimilarity coefficient (GD) for handling mixed data types [16, 17]. GD measures the dissimilarity between a pair of observations, in this case, pairs of studies across multiple aggregate characteristics, with values ranging from 0 (no difference) to 1 (maximum difference).

Consider a network of N studies investigating distinct sets of T treatments, forming a connected network with 2<P≤T2 observed treatment comparisons. From each study, we extract the *same* set of Z characteristics. Typically, some characteristics will be missing across studies. To quantify the dissimilarity among all study pairs in the network, we calculate a N×N symmetric dissimilarity matrix with a zero diagonal using the GD metric. The GD metric, d(x,y), between the studies x and y for a set of Z characteristics is the weighted average of $d{\left(x,y\right)}_{i}$:

(1) $$d\left(x,y\right)=\frac{\sum_{i=1}^{Z}\delta_{\mathrm{xy},i}d{\left(x,y\right)}_{i}}{\sum_{i=1}^{Z}\delta_{\mathrm{xy},i}}$$

with $d{\left(x,y\right)}_{i}$ being the dissimilarity between these two studies for characteristic i and $\delta_{\mathrm{xy},i}$ being a dummy variable that indicates whether characteristic i is observed for both compared studies:

$$\delta_{\mathrm{xy},i}=\left\{\begin{array}{l} 0,\;\text{if}\;x_{i}\;\text{or}\;y_{i}\;\text{is missing}\\ 1,\;\text{if}\;x_{i}\;\text{and}\;y_{i}\;\mathrm{are}\;\text{observed} \end{array}\right.$$

where $x_{i}$ and $y_{i}$ are the values of the characteristic i in the two studies. Therefore, the denominator of d(x,y) equals the number of characteristics observed in both studies.

There are different formulas to calculate $d{\left(x,y\right)}_{i}$ depending on whether the characteristic is quantitative (numeric) or qualitative. For a numeric characteristic i, the dissimilarity between two studies, x and y, is conventionally calculated as follows:

(2) $$d{\left(x,y\right)}_{i}=\frac{\left|x_{i}-y_{i}\right|}{R_{i}}$$

where $x_{i}$ and $y_{i}$ are the values of the characteristic i in the two studies, and $R_{i}$ is the range of the observed values for that characteristic. This results in $d{\left(x,y\right)}_{i}=0$, if $x_{i}=y_{i}$, and $d{\left(x,y\right)}_{i}\in \left(0,1\right]$, if $x_{i}\neq y_{i}$. In the Discussion, we offer our position about routinely using the observed range, which could be sensitive to outliers, as a single outlier could substantially influence the calculation of the GD metric.

For an unordered categorical characteristic i, the dissimilarity between the studies x and y is conventionally calculated as:

$$d{\left(x,y\right)}_{i}=\left\{\begin{array}{l} 1,\;\text{if}\;x_{i}\neq y_{i}\\ 0,\;\text{if}\;x_{i}=y_{i} \end{array}\right.$$

with $x_{i}$, $y_{i}\in \left\{1,2,\ldots ,k\right\}$ for a characteristic with k≥2 unordered categories. Lastly, for an ordered categorical characteristic i, the dissimilarity between the studies x and y, is obtained as follows:

$$d{\left(x,y\right)}_{i}=\frac{\left|\mathrm{rank}\left(x_{i}\right)-\mathrm{rank}\left(y_{i}\right)\right|}{{\mathrm{RR}}_{i}}$$

where $x_{i}$, $y_{i}\in \left\{1,2,\ldots ,k\right\}$ for a characteristic with k>2 ordered categories, $\mathrm{rank}\left(x_{i}\right)$ and $\mathrm{rank}\left(y_{i}\right)$ are the ranked values of the studies x and y, respectively, for the characteristic i and ${\mathrm{RR}}_{i}$ is the range of the ranked values for that characteristic. Similar to the numeric characteristic, $d{\left(x,y\right)}_{i}=0$, if $\mathrm{rank}\left(x_{i}\right)=\mathrm{rank}\left(y_{i}\right)$, and $d{\left(x,y\right)}_{i}\in \left(0,1\right]$, if $\mathrm{rank}\left(x_{i}\right)\neq \mathrm{rank}\left(y_{i}\right)$.

In systematic reviews, numeric characteristics are typically reported using several summary statistics, such as mean with standard deviation or median with minimum and maximum. Incorporating the same characteristic multiple times in Equation (1) would affect the GD metric. Nonetheless, it is not straightforward to defend selecting only one, as different summary statistics for the same numeric characteristic would yield different GD metric values. Therefore, the GD metric between the studies x and y should be modified by introducing characteristic‐specific weights as follows:

(3) $$d\left(x,y\right)=\frac{\sum_{i=1}^{Z}w_{i}\delta_{\mathrm{xy},i}d{\left(x,y\right)}_{i}}{\sum_{i=1}^{Z}w_{i}\delta_{\mathrm{xy},i}}$$

with $w_{i}$ being the weight for characteristic i, which can be defined, for instance, as $w_{i}\in \left[0,1\right]$ with $\sum_{i=1}^{Z}w_{i}=Z$ if all characteristics contribute equally to the GD metric. Equation (1) assigns equal weight to all characteristics. A tentative suggestion for weight would be the following: characteristics included once receive a weight equal to 1, and each summary statistic of a numeric characteristic receives a weight equal to 1 divided by the number of summary statistics considered for that numeric characteristic so that the sum of these weights still gives 1 for that characteristic. We discuss further the need for properly defined weights for the characteristics in the homonymous section. In the subsequent sections, we consider the weighted GD metric.

Supporting Information S2, Methods 1 describes how to deal with missing data when calculating the GD metric. Table 1 summarizes the terminology of the proposed framework as it unfolds in the following sections.

**TABLE 1:** Terminology of the proposed framework as it appears in the article.

Term	Explanation
$d{\left(x,y\right)}_{i}$	The dissimilarity between studies x and y for characteristic i.
d(x,y)	The Gower's dissimilarity coefficient between studies x and y for a set of characteristics and their weights.
{d}∑i=1NTi2×∑i=1NTi2	A symmetric matrix of d(x,y) for all pairs of N studies included in a connected network, with $\left|T_{i}\right|$ being the number of treatments in the study i to account for multi‐arm studies. The main diagonal is zero.
Within‐comparison dissimilarity ($D_{p}^{W}$)	The root mean square dissimilarity of the lower off‐diagonal elements of {d}∑i=1NTi2×∑i=1NTi2 corresponding to the dissimilarities among the studies investigating the treatment comparison p. It quantifies the non‐statistical heterogeneity in the corresponding comparison.
Between‐comparison dissimilarity ($D_{pp^{\prime }}^{B}$)	The root mean square dissimilarity of the lower off‐diagonal elements of {d}∑i=1NTi2×∑i=1NTi2 corresponding to the dissimilarities between the studies investigating comparison p and the studies of comparison $p^{\prime }$. It quantifies the non‐statistical heterogeneity in the corresponding comparison between two treatment comparisons.
${\left\{D\right\}}_{P\times P}$	A (symmetric) dissimilarity matrix for a network of P observed treatment comparisons containing the within‐comparison dissimilarities ($D_{p}^{W}$) in the main diagonal and the between‐comparison dissimilarities ($D_{pp^{\prime }}^{B}$) in the off‐diagonal elements.
Threshold of low dissimilarity	The median of a selected predictive distribution for the $I^{2}$ statistic in a future meta‐analysis that aligns with the investigated outcome type, treatment‐comparator type and average study size in the network. A $D_{p}^{W}$ (or $D_{pp^{\prime }}^{B}$) below the threshold indicates “low” dissimilarity (or likely transitivity in the corresponding part of the network); otherwise, dissimilarity is “likely concerning” (or intransitivity is suspicious).
“Fragmented” comparison	A treatment comparison whose studies are arranged into two or more clusters based on hierarchical agglomerative clustering.

### 3.2 Within‐Comparison and Between‐Comparison Dissimilarities

For a network with N studies, the dissimilarity matrix with GD values is indicated by {d}∑i=1NTi2×∑i=1NTi2, with $\left|T_{i}\right|$ being the number of treatments in study i to account also for the dissimilarity between each comparison of a multi‐arm study and the two‐arm studies; hence, ${\left\{d\right\}}_{N\times N}$ indicates that the network includes only two‐arm studies. The dissimilarity matrix {d}∑i=1NTi2×∑i=1NTi2 quantifies how (dis)similar are the study pairs in a network; however, we need to map this quantity to the P observed comparisons in a network. To do so, for each observed treatment comparison informed by at least two studies, we convert the corresponding GD values in {d}∑i=1NTi2×∑i=1NTi2 into a single number that captures the overall dissimilarity for that comparison. This quantity is referred to as *within‐comparison dissimilarity* ($D_{p}^{W}$) and equals the root mean square of the GD values of the corresponding non‐diagonal elements of {d}∑i=1NTi2×∑i=1NTi2:

DpW=∑jd(x,y)(j)−02h2

where $D_{p}^{W}$ is the overall dissimilarity in comparison p=1,2,…,P with h studies and $d{\left(x,y\right)}_{\left(j\right)}$ is the dissimilarity j=1,2,…,h2 between two studies for that comparison. Essentially, this is the formula for the population standard deviation after replacing the mean with zero, which we preferred to the arithmetic mean as we aimed to measure the spread of the dissimilarities from zero (complete similarity). A single‐study comparison has zero (within‐comparison) dissimilarity for including one study.

To calculate the overall dissimilarity between any two treatment comparisons, we use the same formula as above, considering the dissimilarities between the studies of one treatment comparison versus the studies of the other. This quantity is referred to as *between‐comparison dissimilarity*, denoted as $D_{pp^{\prime }}^{B}$, where p, $p^{\prime }\in \left\{1,2,\ldots ,P\right\}$, $\;p\neq p^{\prime }$ are the compared treatment comparisons.

Essentially, $D_{p}^{W}$ and $D_{pp^{\prime }}^{B}$ reflect the overall non‐statistical heterogeneity (i.e., variability in clinical and methodological characteristics of the studies) observed in the corresponding comparison (e.g., B versus A in all studies investigating this comparison) and comparison between comparisons (e.g., B versus A comparison against C versus A comparison), respectively. Then, the P within‐comparison dissimilarities are arranged at the diagonal of the new matrix, ${\left\{D\right\}}_{P\times P}$, and the P2 between‐comparison dissimilarities populate the off‐diagonal elements of the matrix. For example, for a fully connected network with three treatments, A, B, and C, the dissimilarity matrix ${\left\{D\right\}}_{3\times 3}$ is constructed as follows:

$$D=\left(\begin{array}{lll} D_{\mathrm{BA}}^{W} & D_{\mathrm{BA},\mathrm{CA}}^{B} & D_{\mathrm{BA},\mathrm{CB}}^{B}\\ D_{\mathrm{CA},\mathrm{BA}}^{B} & D_{\mathrm{CA}}^{W} & D_{\mathrm{CA},\mathrm{CB}}^{B}\\ D_{\mathrm{CB},\mathrm{BA}}^{B} & D_{\mathrm{CB},\mathrm{CA}}^{B} & D_{\mathrm{CB}}^{W} \end{array}\right)$$

The dissimilarity matrix ${\left\{D\right\}}_{P\times P}$ is symmetric with elements in the interval [0,1], aligning with the scale of {d}∑i=1NTi2×∑i=1NTi2. Unlike traditional dissimilarity matrices, the diagonal elements of ${\left\{D\right\}}_{P\times P}$ are not zero (they can take any value in [0,1]), except for the case of single‐study comparison(s).

Between‐comparison dissimilarities are crucial for assessing transitivity in a network. Low $D_{pp^{\prime }}^{B}$ suggests minimal imbalances in the distribution of the effect modifiers in the corresponding comparisons, indicating potential transitivity in the corresponding part of the network. For instance, if $D_{\mathrm{CA},\mathrm{BA}}^{B}$ is low, using the summary effects of the BA and CA studies may provide a valid indirect estimate for the CB comparison. Furthermore, if $D_{\mathrm{CB},\mathrm{BA}}^{B}$ and $D_{\mathrm{CB},\mathrm{CA}}^{B}$ are also low, combining the indirect and direct CB effects may lead to a valid and more precise “mixed” effect for CB, demonstrating transitivity in the network. Contrariwise, a high $D_{\mathrm{CA},\mathrm{BA}}^{B}$ suggests substantial imbalances in the distribution of the effect modifiers in the compared comparisons, potentially invalidating the indirect CB effect due to intransitivity. Similarly, if $D_{\mathrm{CB},\mathrm{BA}}^{B}$ and $D_{\mathrm{CB},\mathrm{CA}}^{B}$ are high but $D_{\mathrm{CA},\mathrm{BA}}^{B}$ is low, pooling the valid indirect CB effect with the direct one would yield a meaningless “mixed” effect for CB for mixing different populations and methodologies, rendering this “mixed” effect inappropriate for recommendation [3, 18].

#### 3.2.1 Threshold of Low Dissimilarity

To our knowledge, no empirical study has explored the extent of clinical and methodological inconsistency across studies in various systematic reviews within different healthcare fields. Ideally, establishing a threshold to facilitate a binary decision—in our case, indicating “low” or “likely concerning” dissimilarity—should be based on empirical evidence closely aligned with the investigated condition, population and treatments to mitigate subjectivity. Drawing inspiration from an existing empirical study on the statistical inconsistency across studies, as measured by the $I^{2}$ statistic [19], we *provisionally* adopt the predictive distribution for the inconsistency expected in a future meta‐analysis for a mixed outcome (e.g., binary, continuous, and time‐to‐event data) [20]. The rationale for this decision stems from the fact that $D_{p}^{W}$ and $D_{pp^{\prime }}^{B}$ are defined in the same value range as the $I^{2}$ statistic and result from characteristics with likely mixed data types.

Rhodes et al. [20] generated distinct predictive distributions for $I^{2}$ based on the type of outcome (i.e., all‐cause mortality, semi‐objective and subjective), the treatment‐comparator (i.e., pharmacological vs. placebo/control, pharmacological vs. pharmacological, and any non‐pharmacological treatments), and the average size of the included studies (i.e., less than 50, between 50 and 200 and over 200 participants) expected in a future meta‐analysis. More details regarding the definition of the different outcome types and non‐pharmacological treatments can be found in Rhodes and colleagues [20]. In alignment with Rhodes et al. [20], we may consider different thresholds for “low” dissimilarity based on the aforementioned design factors. Specifically, we define dissimilarity as “low” if a $D_{pp^{\prime }}^{B}$ (or $D_{p}^{W}$) falls below the median of the selected predictive distribution for $I^{2}$; otherwise, dissimilarity is “likely concerning.” Table 2 summarizes the thresholds of “low” dissimilarity for various design factors, including the case of a general healthcare setting.

**TABLE 2:** Threshold of “low” dissimilarity for various design factors.^a^,^b^

Design factor	Pharmacological versus placebo/control	Pharmacological versus pharmacological	Any non‐pharmacological
General healthcare setting	*0.13* (0.0002, 0.99)		
Average study size is below 50 participants			
All‐cause mortality	*0.0007* (< 0.000001, 0.91)	*0.0004* (< 0.000001, 0.76)	*0.0007* (< 0.000001, 0.87)
Semi‐objective	*0.06* (0.00008, 0.97)	*0.04* (0.00009, 0.89)	*0.06* (0.00006, 0.98)
Subjective	*0.25* (0.006, 0.94)	*0.16* (0.01, 0.80)	*0.24* (0.003, 0.97)
Average study size is between 50 and 200 participants			
All‐cause mortality	*0.0007* (< 0.000001, 0.89)	*0.0004* (< 0.000001, 0.74)	*0.0007* (< 0.000001, 0.86)
Semi‐objective	*0.06* (0.00008, 0.97)	*0.04* (0.00007, 0.90)	*0.06* (0.00005, 0.98)
Subjective	*0.25* (0.005, 0.94)	*0.16* (0.01, 0.79)	*0.23* (0.003, 0.97)
Average study size is over 200 participants			
All‐cause mortality	*0.0008* (< 0.000001, 0.91)	*0.0005* (< 0.000001, 0.75)	*0.0007* (< 0.000001, 0.88)
Semi‐objective	*0.07* (0.0001, 0.97)	*0.04* (0.00009, 0.91)	*0.06* (0.00006, 0.98)
Subjective	*0.28* (0.0006, 0.95)	*0.18* (0.01, 0.82)	*0.26* (0.003, 0.97)

^a^ The content results from tables 3 and 8 in [20] and refers to the median and 95% interval (in parenthesis) of the predictive distribution for $I^{2}$ in a future meta‐analysis with mixed outcome data. Tables 3 and 8 in [20] report these numbers in percentages.

^b^ The median of the distribution (in italics) represents the threshold of “low” dissimilarity for the corresponding design factors.

Using this threshold definition and visualizing the dissimilarity matrix ${\left\{D\right\}}_{P\times P}$ with a heatmap, we can color‐code the between‐comparison dissimilarities (the off‐diagonal elements) as green for “low” and red for “likely concerning” dissimilarity, along with the within‐comparison dissimilarities (the main diagonal elements). In the ideal scenario of transitivity, all off‐diagonal elements would be green. If we suspect that part of the network may be intransitive, the heatmap helps detect pairs of dissimilar comparisons that need to be scrutinized for their (extracted) effect modifiers.

### 3.3 Hierarchical Agglomerative Clustering

Hierarchical (agglomerative) clustering is an unsupervised learning method that organizes pairs of observations into a hierarchy of clusters based on a dissimilarity matrix and a linkage method, a metric indicating how close the clusters are. This process creates a dendrogram, a tree‐like graph, that illustrates the nested arrangements of clusters and their distances. Various linkage methods, including single linkage (nearest neighbor), complete linkage (furthest neighbor) and centroid, have been proposed and can be used in this context to group the studies into clusters [12]. Our novel approach involves utilizing the dissimilarity matrix {d}∑i=1NTi2×∑i=1NTi2 as input for the hierarchical clustering. Key decisions include selecting the linkage method and determining the clustering partition (i.e., the number of clusters), which should be guided by objective criteria, like the cophenetic correlation coefficient [21] and the (overall average) silhouette width.

#### 3.3.1 Cophenetic Correlation Coefficient

The cophenetic correlation coefficient (CCC) assesses the goodness‐of‐fit of a linkage method to the dissimilarity matrix (for a selected dissimilarity metric) and falls within the range [−1,1] [21]. Essentially, CCC measures how faithfully a dendrogram preserves the pairwise distances between the original data points. A CCC close to 1 indicates a strong match between the linkage method and the dissimilarities among treatment comparisons. Supporting Information S1, Table S3 summarizes the linkage methods used in this study. The optimal linkage method corresponds to the highest positive CCC value. When multiple linkage methods yield the same high CCC value, we select one randomly.

#### 3.3.2 Silhouette Width

Internal and external validation methods are crucial for objectively determining the optimal number of clusters. In our context, we lack external information about the “true” cluster number; therefore, we rely on internal validity measures to assess the quality of the clustering structure. Several internal validity methods exist, with the most widely recognized ones being the connectivity index, silhouette width, and Dunn index [22, 23]. We consider the silhouette width for its bounded range of values, aiding interpretation. The silhouette width [24] measures cluster compactness (i.e., the distance between the comparisons within the same cluster) and separation (i.e., how well‐separated a cluster is from other clusters). It is calculated per study and ranges from −1 to 1, with values closer to 1 indicating strong partitioning, values near 0 suggesting overlapping clusters, and negative values implying potential misclassification. Further details on the silhouette width can be found in the Supporting Information S2, Methods 2.

#### 3.3.3 Dendrogram of Studies and Their Treatment Comparisons

The resulting classifications from hierarchical clustering are visualized as a hierarchical tree, known as dendrogram, which depicts the nested arrangements of clusters and their distances [12, 21]. The dendrogram is a versatile visualization tool, facilitating an understanding of the clustering process for a chosen linkage method and clustering partition. It is typically presented alongside the heatmap of dissimilarities to aid interpretation. We adopted this approach to visualize the results from clustering, and we color‐coded the branches of the dendrogram to indicate the distinct clusters. The leaves of the dendrogram represent the studies with the corresponding treatment comparisons. The dissimilarity matrix {d}∑i=1NTi2×∑i=1NTi2 was employed to generate the heatmap. However, in extensive networks featuring more than three clusters with a mix of treatment comparisons, a dendrogram integrated with a heatmap could be challenging to interpret. In the next section, we propose a practical solution to address the volume of information in a dendrogram when dealing with large networks.

#### 3.3.4 “Fragmented” Comparisons and Transitivity Evaluation

Hierarchical clustering organizes the studies into two or more clusters, with these clusters likely containing a mix of treatment comparisons. How can these results be utilized to assess transitivity, particularly when dealing with large networks? We propose examining the clusters for “fragmented” comparisons, wherein studies are arranged into multiple clusters. The higher the number of clusters and “fragmented” comparisons, the greater the concerns about imbalanced effect modifiers across the network. A percent stacked bar plot can be employed to identify the “fragmented” comparisons: the *x*‐axis presents the observed treatment comparisons and subgroups differentiated by colors to represent clusters. A “fragmented” comparison will have two or more subgroups, with their height (*y*‐axis) indicating the percentage of studies found in the corresponding clusters.

**FIGURE 2:**
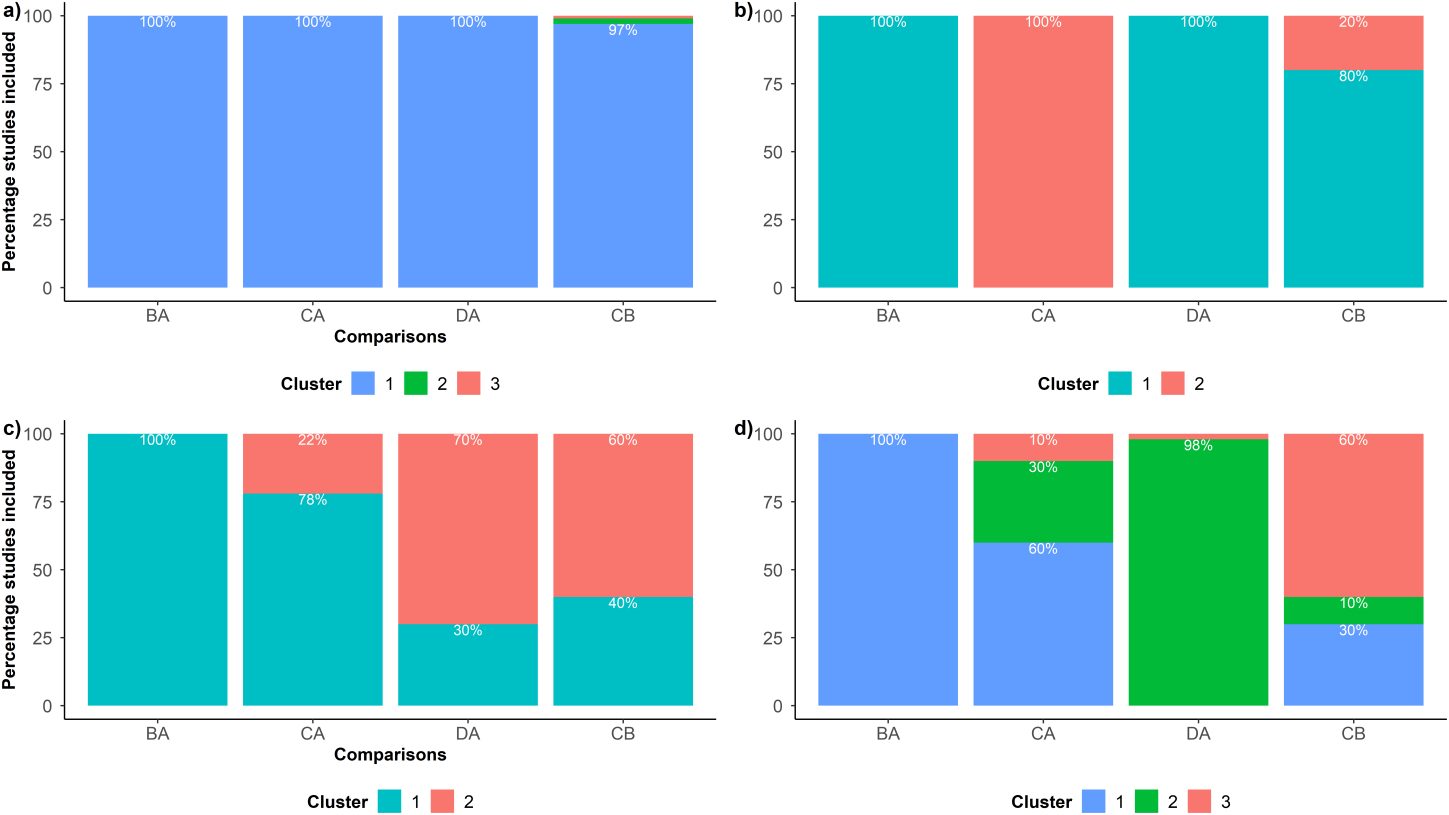
A fictional connected network with four treatment comparisons illustrating the concept of “fragmented” comparisons using four hypothetical clustering scenarios.

The challenge, however, is defining a concerning number of “fragmented” comparisons, which depend on the number of observed treatment comparisons and studies. We present scenarios to guide interpretation illustrated in Figure 2:

- The studies are organized in two or three clusters with few “fragmented” comparisons (e.g., 5% or less), and a small percentage of their studies (e.g., 5% or less) are found in the neighboring cluster(s) (Figure 2a): here, CB is the only “fragmented” comparison with a handful of eloping studies found in the other two clusters. The 5% threshold represents the minimum acceptable percentage of “eloping” studies. While transitivity may be plausible, inspecting the distribution of their effect modifiers is advisable.
- The studies form two clusters, with most comparisons and *all* their studies being found in the same cluster (Figure 2b). Here, hierarchical clustering has largely split the network into two subnetworks with similar comparisons. Transitivity may be plausible for each subnetwork but not for the whole network. In Figure 2b, CB is the unique “fragmented” comparison, with 20% of its studies in the neighboring cluster.
- The studies form two clusters with many “fragmented” comparisons, and many of their studies are found in the neighboring cluster (Figure 2c). While the partitioning is compact, many “fragmented” comparisons imply that they mismatch with most comparisons regarding the distribution of their effect modifiers, raising concerns about the plausibility of transitivity. In Figure 2c, all comparisons, except for BA, are “fragmented” for containing several eloping studies.
- The studies form more than two clusters with many “fragmented” comparisons. In this case, the partitioning becomes loose with many mismatched comparisons. Again, the evidence base should be scrutinized to verify whether concerns about imbalanced effect modifiers and possible imminent intransitivity are valid. Figure 2d shows the “fragmented” comparisons, CA and CB, containing several eloping studies in neighboring clusters. Only a handful of the studies in the “fragmented” comparison DA are found in the neighboring cluster (cluster 3).

### 3.4 Schematic Illustration of the Proposed Approach

**FIGURE 3:**
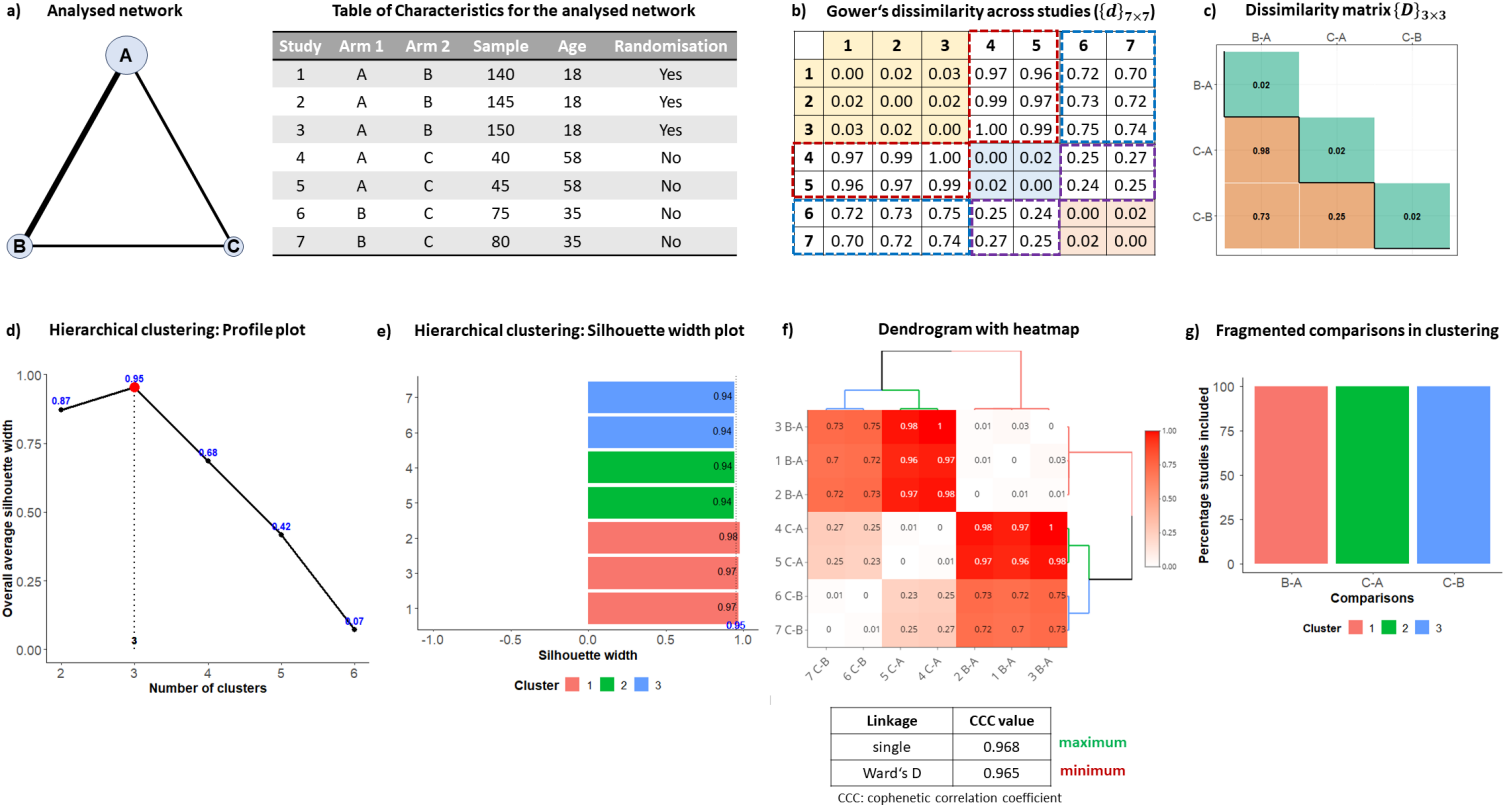
Schematic illustration of the proposed approach to evaluate transitivity using a fictional dataset of seven studies and three characteristics assumed to be effect modifiers.

Figure 3 illustrates our approach to assessing transitivity using a fictional triangle network informed by seven studies investigating three pairwise comparisons: BA, CA, and CB. The accompanying table presents two quantitative study‐level aggregate characteristics (sample size and mean age) and one qualitative (adequate randomization) (Figure 3a). BA is investigated in larger studies with a younger population and adequate randomization, while comparison CA is examined in smaller studies with an older population and inadequate randomization. CB lies between these two comparisons. We anticipate virtually no within‐comparison dissimilarity in all comparisons and substantial dissimilarity between BA and CA, suggesting that they will be in separate clusters.

Initially, we calculated the GD metric for all pairs of studies in the network using Equation (3) with equal weight to all characteristics to generate the dissimilarity matrix ${\left\{d\right\}}_{7\times 7}$ (Figure 3b). The symmetric “submatrices” highlighted in yellow, blue, and red represent the elements used to derive the within‐comparison dissimilarity ($D_{p}^{W}$) for BA, CA, and CB, respectively (Figure 3b). Framed areas in red, blue, and purple contain the elements used to calculate all pairwise dissimilarities $D_{\mathrm{BA},\mathrm{CA}}^{B}$, $D_{\mathrm{BA},\mathrm{CB}}^{B}$, and $D_{\mathrm{CA},\mathrm{CB}}^{B}$ (Figure 3b). The resulting dissimilarity matrix ${\left\{D\right\}}_{3\times 3}$ contains the within‐comparison dissimilarities in the main diagonal and the between‐comparison dissimilarities in the lower off‐diagonal elements (Figure 3c). In this example, we considered a general healthcare setting, defining the threshold of “low dissimilarity” at 0.13 (Table 2). Only the main diagonal (correctly) exhibits “low” dissimilarity (Figure 3c). The between‐comparison dissimilarities highlight “likely concerning” imbalances in the distribution of the three characteristics across comparisons, raising concerns about the validity of their indirect effects (Figure 3c).

In the context of hierarchical clustering, once the dissimilarity matrix ${\left\{d\right\}}_{7\times 7}$ is calculated, we use the profile plot to determine the number of clusters, resulting in a partition of two to six clusters, with three clusters identified as the optimal partitioning (Figure 3d). A silhouette width plot could also be constructed to visualize the silhouette width values for each study based on three clusters (Figure 3e). The dendrogram with integrated heatmap (i.e., ${\left\{d\right\}}_{7\times 7}$) shows all studies of comparison BA forming one cluster, while all studies of comparisons CB and CA formed separate clusters (Figure 3f). Notably, the cluster with CB studies was found to be closer to CA than the BA cluster. The stacked bar plot displayed no “fragmented” comparisons, indicating the correct grouping of all studies into their comparisons and raising concerns about the possibility of transitivity for having three clusters (Figure 3g).

### 3.5 Computational Programs and Data Repository

The rnmamod [25, 26] R package was used to calculate the GD metric, produce the matrices {d}∑i=1NTi2×∑i=1NTi2 and ${\left\{D\right\}}_{P\times P}$, and perform the hierarchical agglomerative clustering for the fictional network and both motivating examples. All figures were created using the synergy of rnmamod and ggplot2 R packages. The functions to replicate the results from both motivating examples are available online at https://github.com/LoukiaSpin/Hierarchical‐Clustering‐Transitivity‐Evaluation.git. The script of the fictional network to construct Figure 3 also aids as a brief tutorial to apply the functions of the rnmamod R package developed for the proposed analysis framework. Supporting Information S2, Methods 3 lists all R packages considered in developing the rnmamod R functions for the present work.

## 4 Results

### 4.1 Network on Rheumatoid Arthritis

The network involved six pairwise treatment comparisons, each comprising at least three studies (Figure 1a; Singh et al. [14]). All 27 studies provided data on 10 study‐level aggregate characteristics without missing information. Figure S1 (Supporting Information S1) shows violin plots illustrating the distribution of two quantitative characteristics: study and disease durations. There were variations in the range of values across the comparisons, particularly for the disease duration, but with some overlap in the distribution overall.

For qualitative characteristics, as depicted in Figure S2 (Supporting Information S1), most comparisons exhibited a consensus in the frequency of their categories, apart from prior drug failure and rheumatoid arthritis duration, which had more than two categories. Anti‐TNF therapies (ADA, ETA, and INF) demonstrated within‐comparison homogeneity due to their common feature across all included studies (Supporting Information S1, Figure S2). In contrast, ABA, ANA, and RIT are not anti‐TNF therapies.

#### 4.1.1 Within‐Comparison and Between‐Comparison Dissimilarity

**FIGURE 4:**
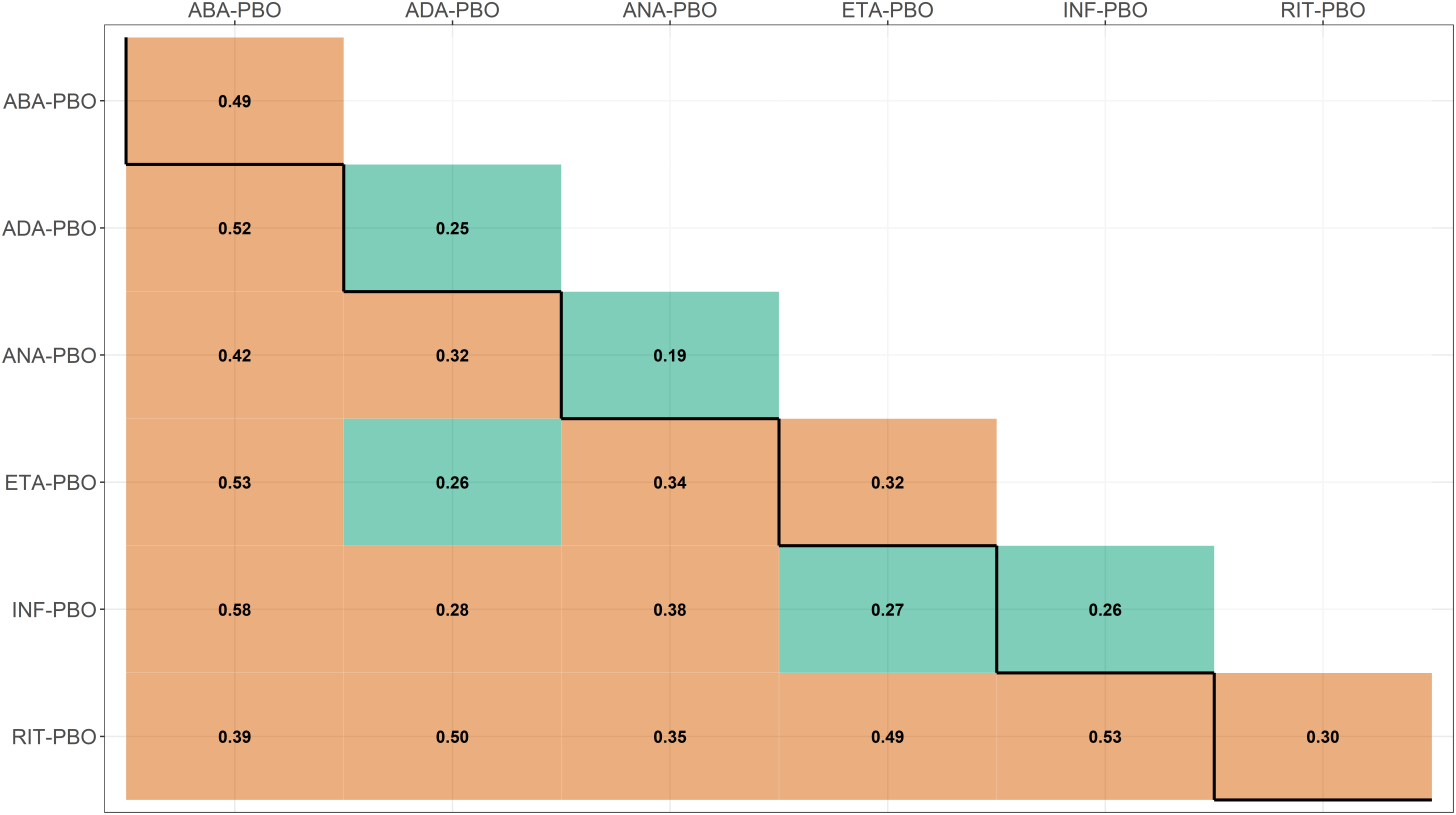
Heatmap on within‐comparison dissimilarities (main diagonal) and between‐comparison dissimilarities (lower off‐diagonal) for the network on rheumatoid arthritis [14]. The row and column names refer to observed treatment comparisons in the network. Green and red cells refer to “low” and “likely concerning” dissimilarities, respectively, based on the threshold of 0.28 for a subjective outcome, comparisons with a placebo and an average sample size of over 200 participants. ABA, abatacept; ADA, adalimumab; ANA, anakinra; ETA, etanercept; INF, infliximab; PBO, placebo; RIT, rituximab.

The network focused on a subjective outcome, including only placebo comparisons, with an average study size of 283 participants. We, thus, set the threshold of “low” dissimilarity at 0.28 (Table 2). In Figure 4, the within‐comparison dissimilarities (main diagonal) indicated that half of the comparisons exhibited “low” $D_{p}^{W}$, ranging from 0.19 (ANA vs. PBO) to 0.26 (INF vs. PBO), suggesting a relatively low non‐statistical heterogeneity across the studies included in these treatment comparisons. The remaining treatment comparisons exhibited a “likely concerning” $D_{p}^{W}$, ranging from 0.30 (RIT vs. PBO) to 0.49 (ABA vs. PBO), suggesting “likely concerning” non‐statistical heterogeneity across their studies (Figure 4).

Between‐comparison dissimilarities were consistently “likely concerning” (Figure 4), ranging from 0.28 (INF‐PBO vs. ADA‐PBO) to 0.58 (INF‐PBO vs. ABA‐PBO). These values signified “likely concerning” imbalances in the distribution of the investigated characteristics, indicating potential intransitivity. Only two inter‐comparisons, ETA‐PBO versus ADA‐PBO and INF‐PBO versus ETA‐PBO, exhibited “low” between‐comparison dissimilarities with $D_{pp^{\prime }}^{B}$ values close to the threshold (0.26 and 0.27, respectively) (Figure 4).

Figure S3 (Supporting Information S1) presents the distribution of the GD values (black points) and the within‐comparison dissimilarities (blue points), whereas Figure S4 (Supporting Information S1) presents the between‐comparison dissimilarities (blue points). The great variability of GD values in all inter‐comparisons justified the substantial values in between‐comparison dissimilarities (Supporting Information S1, Figure S4).

#### 4.1.2 Hierarchical Clustering

The CCC value ranged from 0.86 (using the median linkage method) to 0.88 (using the average linkage method), denoting average as the optimal linkage method for clustering (Supporting Information S1, Table S4). Across a range of two to 26 clusters (for 27 studies), the overall average silhouette width spanned from 0.06 (for 26 clusters) to 0.54 (for two clusters), with two clusters being the optimal partitioning for having the highest overall average silhouette width value (Supporting Information S1, Figure S5). However, it is worth noting that a silhouette width of 0.54 suggests a partitioning of likely moderate compactness and separation. With an increasing number of clusters, the performance of silhouette widths deteriorated, leading to zero silhouette widths, indicating that studies were on the boundary between two neighboring clusters (Supporting Information S1, Figure S6). Eventually, we considered two clusters to color the branches in the dendrogram.

**FIGURE 5:**
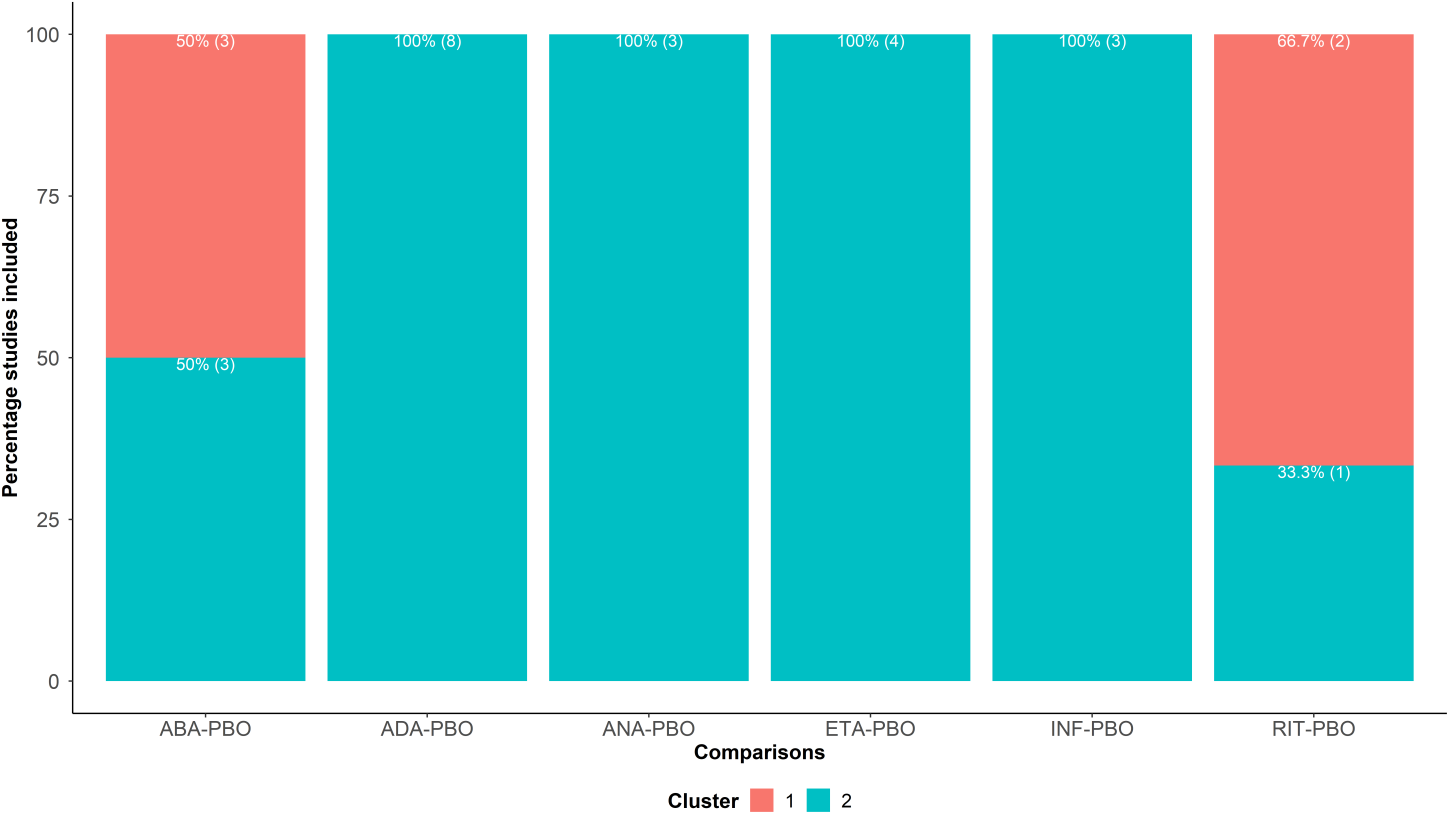
A stacked barplot on “fragmented” comparisons and the corresponding percentage of “eloping” studies based on two clusters indicated by different colors in the bars. Analysis was performed on the network for rheumatoid arthritis [14]. ABA, abatacept; ADA, adalimumab; ANA, anakinra; ETA, etanercept; INF, infliximab; PBO, placebo; RIT, rituximab.

The dendrogram with integrated heatmap revealed two greatly imbalanced clusters in size: the smaller cluster (indicated by red branches) included three studies comparing ABA with PBO and two studies on RIT versus PBO (Supporting Information S1, Figure S7). The neighboring (larger) cluster included the remaining network of studies. Consequently, ABA versus PBO and RIT versus PBO comprised the only “fragmented” comparisons in the network, with most of their studies situated in the small cluster (cluster 1) (Figure 5). The heatmap ${\left\{d\right\}}_{27\times 27}$ showed substantial inter‐cluster dissimilarities, ranging from 0.31 to 0.92 compared to the dissimilarities within each cluster (small cluster: 0.12–0.46, large cluster: 0.01–0.56). However, many intra‐cluster dissimilarities were substantial in both clusters, justifying the challenge of establishing a partitioning with high compactness and separation for this network (Supporting Information S1, Figure S5).

Examining the distribution of characteristics within these two clusters provided interesting insights into the possibility of intransitivity in the network. Comparing the two clusters and drawing conclusions regarding the distribution of quantitative characteristics was challenging due to the severe imbalance in the size of the clusters (Supporting Information S1, Figure S8). However, we identified some noteworthy findings from the qualitative characteristics. Except for the combination of biologic therapy, which was similarly distributed in the two clusters, the clusters mainly comprised different subpopulations (Supporting Information S1, Figure S9). This may raise concerns about the validity of the indirect estimates from this network. The large cluster consisted of studies with predominantly concomitant use of MTX (82%), anti‐TNF biologic (68%), disease‐modifying anti‐rheumatic drugs (DMARDs) as previously failed (91%), late rheumatoid arthritis duration (46%), followed by established (32%) and early rheumatoid arthritis duration (23%), only naïve biologic (100%), and no prior failure of TNF biologic (Supporting Information S1, Figure S9). In contrast, the small cluster did not include any anti‐TNF biologic nor any naïve biologic; furthermore, three studies had no concomitant use of MTX and only biologic as prior failed drugs.

### 4.2 Network on COPD

The network on COPD comprised 39 studies (29 two‐arm, 4 three‐arm, and 6 four‐arm studies) forming 16 pairwise comparisons (Figure 1b), of which two, TIO versus FLU+ and TIO versus FOR, were single‐study comparisons (Baker et al. [15]). In total, 11 study and participant characteristics were recorded across the 39 studies. Figure S10 (Supporting Information S1) presents violin plots illustrating the distribution of quantitative characteristics. Except for maximum FEV1, some comparisons reported identical values across their studies, indicating a high degree of similarity, resulting in strand‐line violin plots. The remaining comparisons exhibited substantial overlap in all characteristics except for maximum FEV1 (Supporting Information S1, Figure S10). Figure S11 (Supporting Information S1) presents the bar plots illustrating qualitative characteristics, indicating a consensus across the comparisons. All studies had adequate random allocation and double blinding. Almost all comparisons included studies with a withdrawal description, except for TIO versus PBO, which included one study without a withdrawal description.

The investigated network had a low amount of missing data across characteristics and studies, comprising only 3.54% of the dataset. Among these characteristics, FVC at inclusion, smoking history, and minimum and maximum mean FEV1 had more missing data, ranging from 7.79% to 9.09% (Supporting Information S1, Figure S12). Additionally, FEV1 at inclusion (two studies) and withdrawal description (one study) also showed some missing data, as illustrated in Figure S12 (Supporting Information S1). No missing data were found for the remaining characteristics. Figures S13 and S14 shed light on the comparisons and their studies contributing to the missing data. Specifically, placebo comparisons involving FLU, FLU+, SAL, and TIO, as well as the comparison of TIO versus SAL, included at least one study that did not report two or more of the abovementioned characteristics. FVC at inclusion was missing in the single‐study comparison of TIO versus FLU+, as shown in Figures S13 and S14.

#### 4.2.1 Within‐Comparison and Between‐Comparison Dissimilarity

**FIGURE 6:**
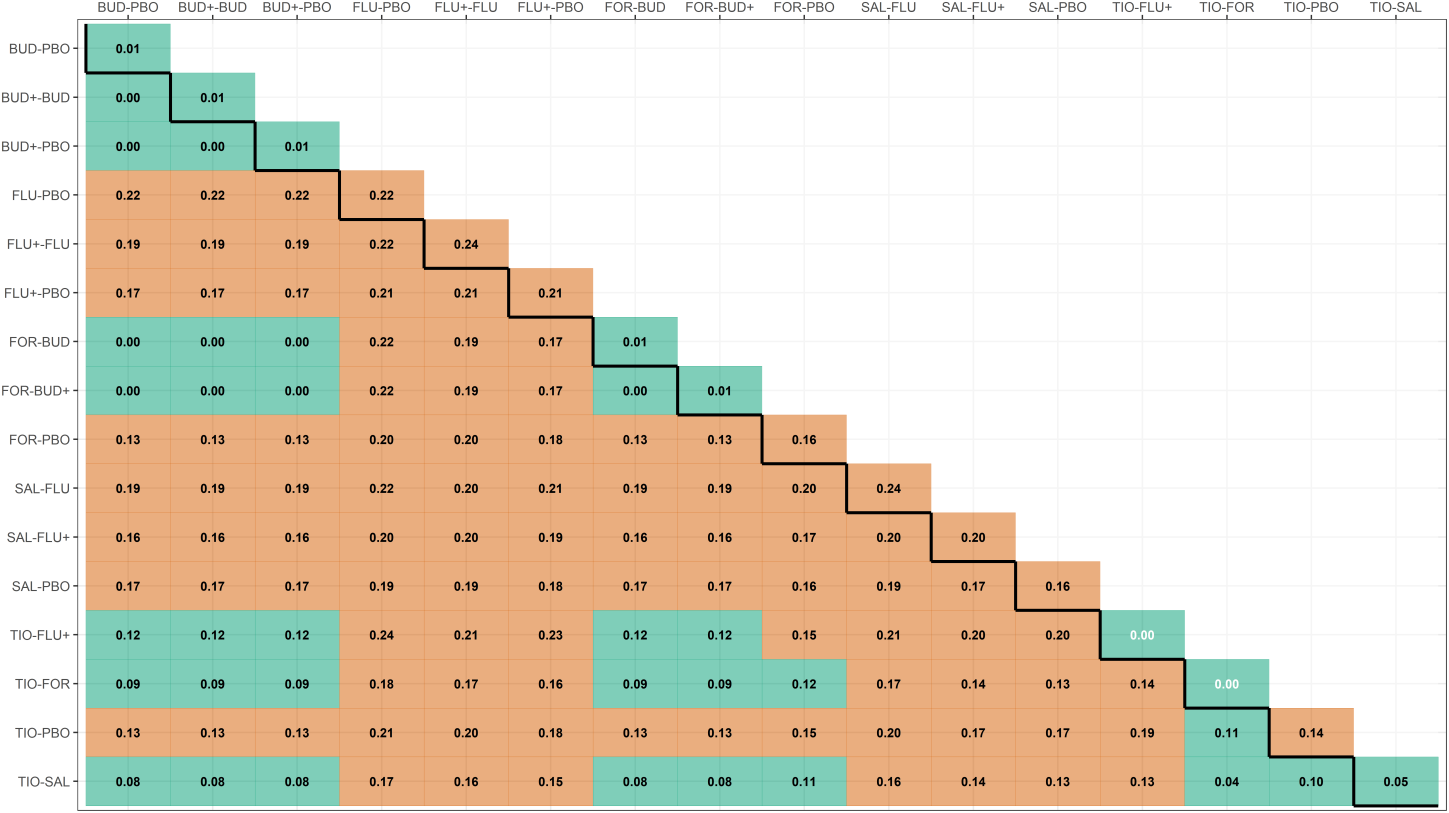
Heatmap on within‐comparison dissimilarities (main diagonal) and between‐comparison dissimilarities (lower off‐diagonal) for the network on chronic obstructive pulmonary disease [15]. The row and column names refer to observed comparisons in the network. Green and red cells refer to “low” and “likely concerning” dissimilarities, respectively, based on the threshold of 0.13 for a general healthcare setting. BUD, budesonide; BUD+, budesonide plus formoterol; FLU, fluticasone; FLU+, fluticasone plus salmeterol; FOR, formoterol; PBO. placebo; SAL, salmeterol; TIO, tiotropium.

We assigned a weight of 0.5 to minimum and maximum FEV1 to account for using two summary statistics for the same numeric characteristic; the remaining characteristics received a weight of 1 (as conventionally). The definition of COPD exacerbation varied across the studies (table 1 in [15]), making it challenging to determine whether the outcome was purely semi‐subjective or subjective. To address this, we selected the threshold at 0.13 (referring to the general healthcare setting) as a compromise between the different outcome types (Table 2). Contrary to the network on rheumatoid arthritis, the COPD network exhibited lower dissimilarities overall, ranging from 0.01 to 0.24, signaling a lower nonstatistical heterogeneity within and across comparisons (Figure 6).

TIO versus SAL and the small subnetwork (with two four‐arm studies and two two‐arm studies containing PBO, BUD, BUD+, and FOR) showed nearly zero within‐comparison dissimilarity, except for the comparison FOR versus PBO (Figure 6). Thirty (25%) of the between‐comparison dissimilarities were considered “low” for not exceeding the threshold. These findings were specific to comparisons among the treatments forming the small subnetwork (excluding FOR versus PBO) and comparisons of the small subnetwork with TIO versus FLU+, TIO versus FOR, and TIO versus SAL, as well as among TIO versus SAL, TIO versus FOR, and TIO versus PBO (Figure 6).

It is important to note that if we were confident that the outcome was subjective, for an average study size of 674 participants, the threshold would have been 0.28, indicating “low” within‐comparison and between‐comparison dissimilarities in the whole network. Figures S15 and S16 present the distribution of the GD values alongside the within‐comparison and between‐comparison dissimilarities, respectively.

#### 4.2.2 Hierarchical Clustering

Based on the CCC values, ranging from 0.60 (using the complete linkage method) to 0.82 (using the average linkage method), the average linkage method was identified as optimal for clustering (Supporting Information S1, Table S5). Finding the optimal partitioning for the studies was challenging, as the profile plot highlighted 31 clusters as optimal, with zero silhouette widths for 20 studies (results not shown) and an overall average silhouette width of 0.68, indicating moderate compactness and separation for the formed clusters (Supporting Information S1, Figure S17). A smaller partitioning of two to five clusters yielded even lower overall average silhouette width values, ranging from 0.38 to 0.47 (Supporting Information S1, Figure S17). Examining the silhouette plots for two to five clusters showed that choosing two clusters resulted in misclassifying one study (negative silhouette width) and forming two severely imbalanced clusters (Supporting Information S1, Figure S18a), whereas choosing three clusters did not improve the performance of the silhouette widths overall (Supporting Information S1, Figure S18b). Choosing four or five clusters resulted in several misclassifications, rendering these partitions inappropriate (Supporting Information S1, Figures S18c and S18d). Therefore, we proceeded with two clusters to color the dendrogram branches.

**FIGURE 7:**
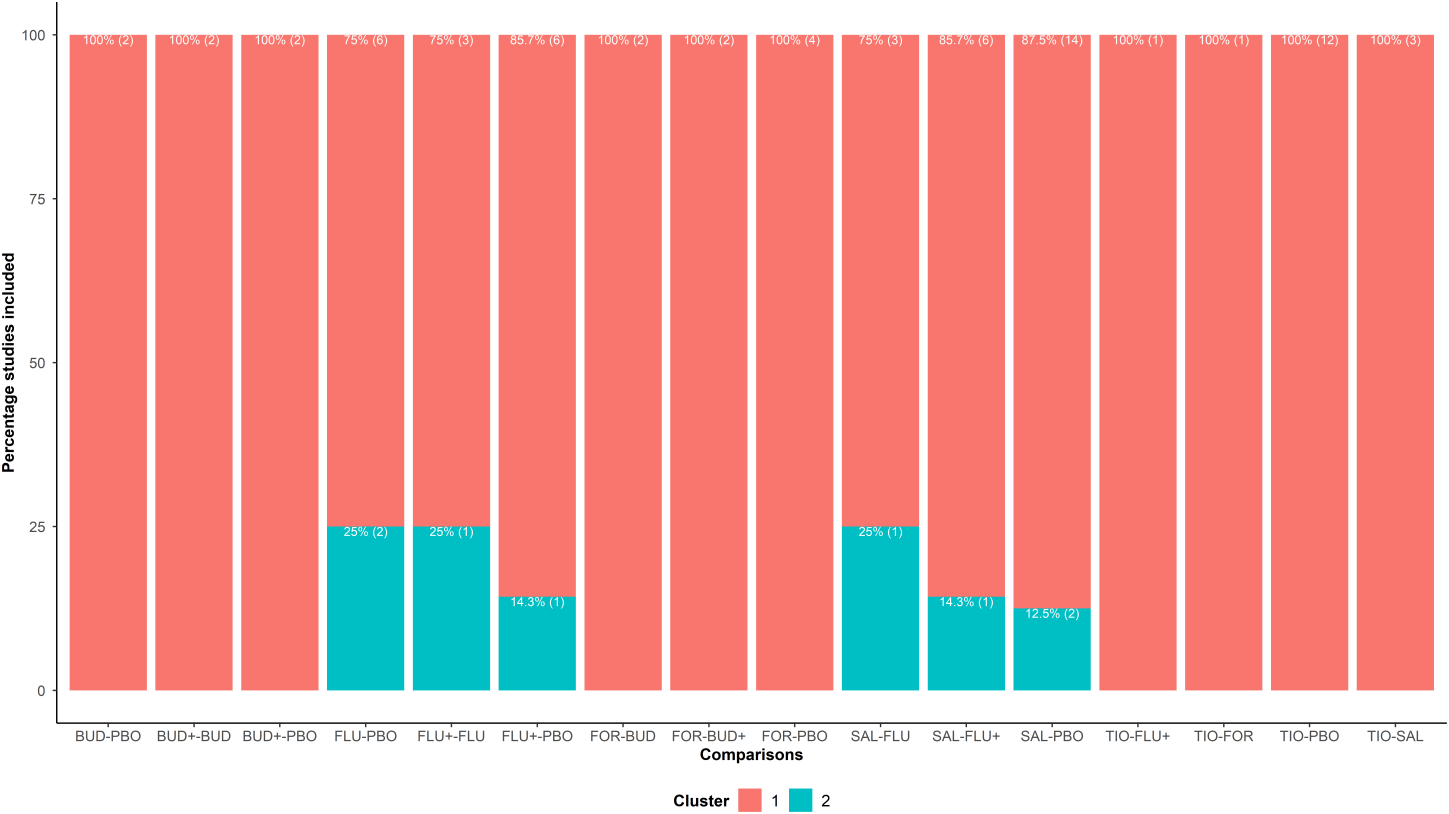
A stacked barplot on “fragmented” comparisons and the corresponding percentage of “eloping” studies based on two clusters indicated by different colors in the bars. Analysis was performed on the network for chronic obstructive pulmonary disease [15]. BUD, budesonide; BUD+, budesonide plus formoterol; FLU, fluticasone; FLU+, fluticasone plus salmeterol; FOR, formoterol; PBO, placebo; SAL, salmeterol; TIO, tiotropium.

Figure S19 (Supporting Information S1) presents the dendrogram with the integrated heatmap ${\left\{d\right\}}_{77\times 77}$ for two clusters, with 90% of the evidence concentrated in one cluster. Six comparisons with up to 25% eloping studies were “fragmented” (Figure 7) and found in the subnetwork formed by PBO, FLU+, SAL, and FLU. The eloping studies pertained to one of the four multi‐arm studies informing the subnetwork, one study on FLU versus PBO, and SAL versus PBO.

Examining the distribution of the characteristics in these two clusters revealed several insights. The larger cluster (cluster 1) included studies with a wider range of study duration, quality score, inclusion FEV1, smoking history, and minimum and maximum FEV1 (%) compared to the smaller cluster (cluster 2) regarding these characteristics (Supporting Information S1, Figure S20). Regarding the qualitative characteristics, all clusters had adequate random allocation, double blinding, and withdrawal description, apart from one study in cluster 1 that did not provide the latter (Supporting Information S1, Figure S21). Hence, we may conclude that the two clusters represented two different subpopulations to some extent for most characteristics (especially the quantitative ones).

## 5 Discussion

The present study introduced study dissimilarities and hierarchical clustering as methods to assess transitivity transparently and enable semi‐objective inferences. Clustering, a well‐established tool for unsupervised machine learning, aligns with the objective of transitivity evaluation by determining whether treatment comparisons are similar enough in terms of effect modifiers to be grouped into one cluster, indicating evidence of possible transitivity. Alternatively, if treatment comparisons should be in several clusters (“fragmented” comparisons), further investigation is needed to assess NMA feasibility.

Both motivating examples suggested potential intransitivity in the networks, indicated by the heatmap of within‐comparison and between‐comparison dissimilarities and the “fragmented” comparisons with sufficient eloping studies. However, evidence of intransitivity does not necessarily indicate inconsistency, particularly if there is low power to test inconsistency [9]. Similarly, applying meta‐regression to increase the plausibility of transitivity may be challenging due to limited variation in covariate values across studies or a small number of studies in the comparisons, hindering the estimation of a statistically significant regression coefficient. When intransitivity is suspected, analysts should first check for extraction errors [3] and investigate measurement scale variability, which can lead to spurious dissimilarities and intransitivity. Consulting clinical epidemiologists to assess whether eloping studies could be merged into one cluster after closely examining the heatmap {d}∑i=1NTi2×∑i=1NTi2 and the distribution of the characteristics per cluster is advised. Alternatively, analysts may consider splitting the network into subnetworks based on clusters or refraining from synthesizing studies if NMA feasibility is questionable. Dias et al. [27] offer valuable insights into addressing inconsistency, which can also be applied in the context of transitivity.

Transitivity is fundamentally an untestable assumption, as its validity relies on epidemiological grounds, challenging the objective assessment of this assumption [3, 28]. The lack of an established evaluation framework, in contrast to the extensive literature discussing the conceptual aspects and importance of transitivity in NMA, attests to the complexity of the concept of transitivity. The proposed framework aims to initiate a methodological foundation for transitivity assessment, addressing limitations associated with subjective graphical evaluations and multiple testing in consideration of the reporting and methodological constraints in the published literature.

Like statistical heterogeneity, heterogeneity in clinical and methodological characteristics among collected studies should be anticipated and appropriately assessed. Analysts must identify parts of the treatment network associated with imbalances in the distribution of important effect modifiers, potentially compromising the validity of the indirect effects. Our novel approach signals the studies and their comparisons that deviate from the overall evidence base, facilitating the exploration of questionable transitivity. This framework could also be expanded to detect characteristics contributing to network parts with potential transitivity concerns. Investigating the robustness of NMA results by excluding studies with substantial dissimilarities can be considered, provided network connectivity is maintained. Alternatively, a weighting approach based on study dissimilarities can be applied, assigning less weight to “questionable” studies to contribute less to the summary results. This approach is superior to adjusting for possible intransitivity through network meta‐regression (current status quo), as moderator analysis with aggregate data commonly suffers from limited studies and ecological bias [29]. Addressing potential intransitivity through network meta‐regression would require separate analysis for each effect modifier—as a multivariable model would not be possible due to limited studies, increasing the type I error.

We advocate employing the weighted GD metric (Equation 3) to prevent numeric characteristics summarized by multiple statistics from disproportionately influencing the GD metric. However, there are additional reasons to prefer the weighted over the conventional GD metric (Equation 1). Not all characteristics are equally relevant for the transitivity evaluation [30]: those more influential or likely correlated with others should receive greater weights. Conversely, characteristics with extensive missing data or inconsistent reporting—necessitating transformation to a common “scale,” if feasible—should be down‐weighted. Rather than defining weights a priori, they could be estimated to identify the most influential characteristics, aiding scrutiny for extraction errors or measurement variability. Lastly, we defined weights as ranging from 0 to 1 for each characteristic, ensuring that when all Z characteristics are equally important, then $\sum_{i=1}^{Z}w_{i}=Z$. Alternatively, weights could be specified as percentage contributions to d(x,y) so that $\sum_{i=1}^{Z}w_{i}=1$. Further research is needed to refine weight specification or estimation for more effective transitivity evaluation using the GD metric.

We considered the standard formulas to calculate $d{\left(x,y\right)}_{i}$ based on the characteristic type. Modifications to these formulas have been proposed and examined in simulation studies, where the analysis unit was the participant [31, 32]. These modifications and their properties may also be relevant to the aggregate data, where the study is the analysis unit. However, calculating dissimilarities based on aggregate characteristics from published clinical studies presents several challenges. A key issue is the missing characteristics and inconsistent reporting of the characteristics across the studies. Another is the extent to which the study characteristics align with the PICO criteria of the systematic review. For example, suppose we have a few qualitative (yes/no) characteristics alongside mean age, which varies narrowly between 60 and 65 across all studies. If participants over 60 were an inclusion criterion of the systematic review, and this range represents the observed values, Equation (2) would calculate a dissimilarity of one between studies with mean ages of 60 and 65. This implies that the age difference is as large as the contrast between yes and no for a qualitative characteristic, giving age undue influence on the GD metric. A possible solution is to base the mean age range on the inclusion criteria of the systematic review rather than the observed study values. Further research is needed to evaluate the GD metric in systematic reviews and propose modifications addressing limitations arising from aggregate data.

How the aggregate characteristics have been extracted is crucial for the credibility of our approach, particularly focusing on participant rather than study design characteristics. The target participant characteristics should be extracted for the whole study, not per treatment arm. This ensures consistency in assessing effect modification, where the association between the outcome and compared treatments may vary based on different levels or values of the effect modifier. When participant characteristics are summarized per arm (e.g., mean age), analysts may average these values using a weighted average, with arm sample sizes comprising the weights. However, guidance on dealing with summarized characteristics per treatment arm is outside the scope of this study and would require further empirical investigation to address extraction challenges related to transitivity assessment.

We proposed using the empirically‐driven predictive distributions of $I^{2}$ to specify thresholds of “low” dissimilarity; however, this is a tentative suggestion necessitated by the lack of an empirical study on the extent of non‐statistical heterogeneity across studies in different healthcare fields. Nonetheless, analysts who wish to consider other thresholds of “low” dissimilarity should explicitly report the process behind the threshold development. The threshold would need to be sensitive to the target population and interventions, as different fields and research questions may have varying sources of non‐statistical heterogeneity.

While our approach provides a quantitative evaluation of transitivity, it is not equipped to handle purely textual data or a combination of quantitative, qualitative, and textual information on the target participants and treatments. This includes a textual description of the inclusion criteria and detailed dose and dosage information, which are crucial for assessing treatment comparison similarity. In such cases, making informed judgments about the plausibility of transitivity may necessitate a qualitative synthesis of the information involving clinical epidemiologists and other content experts, potentially introducing some degree of subjectivity. Prioritizing research on using a combination of qualitative and quantitative synthesis methods to evaluate transitivity is essential in the research agenda for evidence synthesis methods.

## 6 Conclusions

We have developed a novel framework that enables transparent and quantitative exploration of transitivity based on the reported study‐level aggregate characteristics, acting as effect modifiers. All functionalities of the proposed framework are available in the *rnmamod* R‐package, making it easily applicable to a newly planned or published systematic review [26]. For small networks like Singh et al. [14], analysts may present the heatmap‐dendrogram combination and the heatmap of within‐comparisons and between‐comparison dissimilarities in the manuscript. For larger networks, analysts may report the heatmap of within‐comparisons and between‐comparison dissimilarities and the stacked bar plot of “fragmented” comparison in the manuscript and move the heatmap‐dendrogram combination to the appendix. Regardless of the network's size, profile plots on overall average silhouette widths and the silhouette width plot(s) should be reported in the appendix to aid transparency on how the optimal partitioning was determined.

In the absence of external information, the profile plot merely suggests the optimal partition of the studies; however, this optimal choice may lead to information loss when more “compact” clusters are suggested (e.g., Figure S6 in Supporting Information S1). Therefore, we recommend that the analysts consider the first two or three optimal partitions to elucidate whether increasing the number of clusters may lead to material differences in the characteristics' distribution across the clusters, assisting the analysts in a smooth‐running examination of the evidence base when checking the study characteristics by cluster. Analysts should present and meticulously describe the steps taken to address potential intransitivity.

## Author Contributions

L.M.S. conceived the study. L.M.S., K.P., and C.K. designed the study. L.M.S. obtained the data, performed the analyses, and drafted the manuscript. L.M.S., K.P., and C.K. revised the manuscript, interpreted the results, and critically reviewed the manuscript for intellectual content. L.M.S. produced the final version of the submitted article, and K.P. and C.K. approved it.

## Conflicts of Interest

The authors declare no conflicts of interest.

## Supporting information

Supporting Information **S1.**

Supporting Information **S2.**

## Data Availability

The data that support the findings of this study are openly available in the relevant cited publications and are also provided in the Supporting Information of the present article. The functions to replicate the results from both motivating examples are available online at https://github.com/LoukiaSpin/Hierarchical‐Clustering‐Transitivity‐Evaluation.git.
