# Supplementary material for: Exploring the Transitivity Assumption in Network Meta‐Analysis: A Novel Approach and Its Implications
Source: Stat Med. 2025 Apr 10;44(7):e70068. doi: 10.1002/sim.70068 (PMC11983674; doi:10.1002/sim.70068)
Supplement: Supplementary file 1 — Supporting Information S1. [file SIM-44-0-s002.docx]

**Supporting Information for the *Tables and Figures* in the article 'Exploring the transitivity assumption in network meta-analysis: A novel approach and its implications'**

Loukia M. Spineli^1^  [Spineli.Loukia@mh-hannover.de](mailto:Spineli.Loukia@mh-hannover.de)

Katerina Papadimitropoulou^2^* [katerina.papadimitropoulou@amaris.com](mailto:katerina.papadimitropoulou@amaris.com)

Chrysostomos Kalyvas^3^* [chrysostomos.kal@gmail.com](mailto:chrysostomos.kal@gmail.com)

^1^Midwifery Research and Education Unit, Hannover Medical School, Hannover, Germany

^2^Health Economics and Market Access, Amaris Consulting, Lyon, France

^3^Biostatistics and Medical Informatics, Medical Faculty, University of Ljubljana, Vrazov trg 2, 1000 Ljubljana, Slovenia

*Contributed equally

**Supplementary Tables**

**Table S1. Studies and characteristics of the rheumatoid arthritis network (Table 1 in** **Singh et al.)**

| **Studies** | **Arm 1** | **Arm 2** | **Sample size** | **Duration (months)** | **Disease duration**  **(years)** | **MTX use** | **RA duration** | **anti-TNF** | **Prior drugs failed** | **Prior failure TNF** | **Comb. biologic** | **Biologic naive** |
| --- | --- | --- | --- | --- | --- | --- | --- | --- | --- | --- | --- | --- |
| Moreland 2002 | PBO | ABA | 64 | 3 | 3.3 | no | established | no | both | yes | yes | no |
| Genovese 2005 | PBO | ABA | 389 | 6 | 11.9 | no | late | no | biologic | yes | yes | no |
| Schiff 2008 | PBO | ABA | 266 | 6 | 8.1 | yes | established | no | DMARDs | no | yes | yes |
| Kremer 2003 | PBO | ABA | 234 | 6 | 9.2 | yes | established | no | DMARDs | no | yes | yes |
| Kremer 2006 | PBO | ABA | 638 | 12 | 8.6 | yes | established | no | DMARDs | no | yes | yes |
| Weinblatt 2007 | PBO | ABA | 121 | 12 | 12.9 | no | late | no | biologic | yes | no | no |
| Bejarano 2008 | PBO | ADA | 148 | 13 | 0.9 | yes | early | yes | DMARDs | no | yes | yes |
| Breedveld 2006 | PBO | ADA | 525 | 24 | 0.7 | yes | early | yes | DMARDs | no | yes | yes |
| Furst 2003 | PBO | ADA | 531 | 6 | 10.4 | no | late | yes | DMARDs | no | yes | yes |
| Keystone 2004 | PBO | ADA | 407 | 12 | 11 | yes | late | yes | DMARDs | no | yes | yes |
| Kim 2007 | PBO | ADA | 128 | 6 | 6.8 | yes | established | yes | DMARDs | no | yes | yes |
| Miyasaka 2008 | PBO | ADA | 178 | 6 | 9.8 | no | established | yes | DMARDs | no | yes | yes |
| Van De Putte 2004 | PBO | ADA | 223 | 6 | 11.1 | no | late | yes | DMARDs | no | yes | yes |
| Weinblatt 2003 | PBO | ADA | 129 | 6 | 11.7 | yes | late | yes | DMARDs | no | yes | yes |
| Cohen 2002 | PBO | ANA | 153 | 6 | 7.8 | yes | established | no | DMARDs | no | yes | yes |
| Cohen 2004 | PBO | ANA | 501 | 6 | 10.5 | yes | late | no | DMARDs | no | yes | yes |
| Genovese 2004 | PBO | ANA | 161 | 6 | 10.2 | yes | late | no | DMARDs | no | no | yes |
| Moreland 1999 | PBO | ETA | 158 | 6 | 11.5 | no | late | yes | DMARDs | no | yes | yes |
| Weinblatt 1999 | PBO | ETA | 89 | 6 | 13 | yes | late | yes | DMARDs | no | yes | yes |
| COMET 2008 | PBO | ETA | 499 | 12 | 0.9 | yes | early | yes | none | no | yes | yes |
| TEMPO 2004 | PBO | ETA | 459 | 12 | 6.8 | yes | established | yes | DMARDs | no | yes | yes |
| ASPIRE 2004 | PBO | INF | 625 | 12 | 0.8 | yes | early | yes | DMARDs | no | yes | yes |
| ATTRACT 2000 | PBO | INF | 174 | 12 | 10.5 | yes | late | yes | DMARDs | no | yes | yes |
| Quinn 2005 | PBO | INF | 20 | 12 | 0.7 | yes | early | yes | none | no | yes | yes |
| Edwards 2004 | PBO | RIT | 80 | 6 | 11.5 | yes | late | no | DMARDs | no | yes | yes |
| DANCER 2006 | PBO | RIT | 244 | 6 | 10.1 | yes | late | no | both | yes | yes | no |
| REFLEX 2006 | PBO | RIT | 499 | 6 | 11.9 | yes | late | no | biologic | yes | yes | no |

ABA, abatacept; ADA, adalimumab; ANA, anakinra; Comb. biologic, combination biologic therapy; DMARDs, disease-modifying antirheumatic drug; ETA, etanercept; INF, infliximab; MTX use, concomitant use of MTX; PBO, placebo; RA, rheumatoid arthritis; RIT, rituximab.

**Table S2. Studies and characteristics of the chronic obstructive pulmonary disease network (Tables 1 and 2 in** **Baker et al.)**

| **Studies** | **Arm 1** | **Arm 2** | **Sample size** | **Duration** | **Random alloc** | **Double blinding** | **Descr withdraw** | **Quality score** | **Inclusion FEV_1_** | **Inclusion FVC** | **Smoking history** | **Minimum FEV_1_** | **Maximum FEV_1_** |
| --- | --- | --- | --- | --- | --- | --- | --- | --- | --- | --- | --- | --- | --- |
| Llewellyn-Jones 1996 | PBO | FLU | 16 | 8 | yes | yes | yes | 3 | NA | NA | NA | NA | NA |
| Boyd 1997 | PBO | SAL | 456 | 16 | yes | yes | yes | 3 | 70 | 60 | NA | NA | NA |
| Paggiaro 1998 | PBO | FLU | 281 | 24 | yes | yes | yes | 4 | 90 | 70 | 10 | 55 | 59 |
| Mahler 1999 | PBO | SAL | 278 | 156 | yes | yes | yes | 4 | 100 | 70 | 10 | NA | NA |
| Casaburi 2000 | PBO | TIO | 470 | 13 | yes | yes | yes | 4 | 65 | 70 | 10 | 38,6 | 38,6 |
| Burge 2000 | PBO | FLU | 742 | 144 | yes | yes | yes | 5 | 85 | 70 | NA | 50 | 50,3 |
| Littner 2000 | PBO | TIO | 68 | 4 | yes | yes | yes | 4 | 65 | 70 | 10 | 40,5 | 43,4 |
| van Noord 2000 | PBO | SAL | 97 | 12 | yes | yes | yes | 4 | 75 | NA | 10 | 38 | 42 |
| Rennard 2001 | PBO | SAL | 267 | 12 | yes | yes | yes | 4 | 65 | 70 | NA | NA | NA |
| Casaburi 2002 | PBO | TIO | 921 | 52 | yes | yes | yes | 4 | 65 | 70 | 10 | 38,1 | 39,1 |
| Chapman 2002 | PBO | SAL | 408 | 24 | yes | yes | yes | 4 | 85 | 70 | 10 | 44 | 46 |
| Donohue 2002 | PBO | TIO | 410 | 24 | yes | yes | yes | 4 | 60 | 70 | 10 | NA | NA |
| Donohue 2002 | SAL | TIO | 422 | 24 | yes | yes | yes | 4 | 60 | 70 | 10 | NA | NA |
| Donohue 2002 | PBO | SAL | 414 | 24 | yes | yes | yes | 4 | 60 | 70 | 10 | NA | NA |
| Hattotuwa 2002 | PBO | FLU | 30 | 12 | yes | yes | yes | 4 | 80 | NA | 20 | 45,5 | 46,2 |
| Mahler 2002 | PBO | FLU | 349 | 24 | yes | yes | yes | 4 | 56 | 70 | 20 | 40 | 41 |
| Mahler 2002 | FLU | SAL | 328 | 24 | yes | yes | yes | 4 | 56 | 70 | 20 | 40 | 41 |
| Mahler 2002 | FLU | FLU+ | 333 | 24 | yes | yes | yes | 4 | 56 | 70 | 20 | 40 | 41 |
| Mahler 2002 | PBO | SAL | 341 | 24 | yes | yes | yes | 4 | 56 | 70 | 20 | 40 | 41 |
| Mahler 2002 | FLU+ | SAL | 325 | 24 | yes | yes | yes | 4 | 56 | 70 | 20 | 40 | 41 |
| Mahler 2002 | PBO | FLU+ | 346 | 24 | yes | yes | yes | 4 | 56 | 70 | 20 | 40 | 41 |
| Rossi 2002 | PBO | FOR | 645 | 52 | yes | yes | yes | 4 | 70 | 89 | 10 | 46 | 49 |
| Brusasco 2003 | PBO | TIO | 802 | 24 | yes | yes | yes | 4 | 65 | 70 | 10 | 37,7 | 39,2 |
| Brusasco 2003 | SAL | TIO | 807 | 24 | yes | yes | yes | 4 | 65 | 70 | 10 | 37,7 | 39,2 |
| Brusasco 2003 | PBO | SAL | 805 | 24 | yes | yes | yes | 4 | 65 | 70 | 10 | 37,7 | 39,2 |
| Carverley-ERJ 2003 | PBO | BUD | 513 | 52 | yes | yes | yes | 3 | 50 | 70 | 10 | 36 | 36 |
| Carverley-ERJ 2003 | BUD | FOR | 512 | 52 | yes | yes | yes | 3 | 50 | 70 | 10 | 36 | 36 |
| Carverley-ERJ 2003 | BUD | BUD+ | 511 | 52 | yes | yes | yes | 3 | 50 | 70 | 10 | 36 | 36 |
| Carverley-ERJ 2003 | PBO | FOR | 511 | 52 | yes | yes | yes | 3 | 50 | 70 | 10 | 36 | 36 |
| Carverley-ERJ 2003 | BUD+ | FOR | 509 | 52 | yes | yes | yes | 3 | 50 | 70 | 10 | 36 | 36 |
| Carverley-ERJ 2003 | PBO | BUD+ | 510 | 52 | yes | yes | yes | 3 | 50 | 70 | 10 | 36 | 36 |
| Carverley-L 2003 | PBO | FLU | 735 | 52 | yes | yes | yes | 5 | 70 | 70 | 10 | 44,2 | 45 |
| Carverley-L 2003 | FLU | SAL | 746 | 52 | yes | yes | yes | 5 | 70 | 70 | 10 | 44,2 | 45 |
| Carverley-L 2003 | FLU | FLU+ | 732 | 52 | yes | yes | yes | 5 | 70 | 70 | 10 | 44,2 | 45 |
| Carverley-L 2003 | PBO | SAL | 733 | 52 | yes | yes | yes | 5 | 70 | 70 | 10 | 44,2 | 45 |
| Carverley-L 2003 | FLU+ | SAL | 730 | 52 | yes | yes | yes | 5 | 70 | 70 | 10 | 44,2 | 45 |
| Carverley-L 2003 | PBO | FLU+ | 719 | 52 | yes | yes | yes | 5 | 70 | 70 | 10 | 44,2 | 45 |
| Celli 2003 | PBO | SAL | 824 | 12 | yes | yes | yes | 5 | 70 | 65 | 15 | 42,1 | 43,6 |
| Hanania 2003 | PBO | FLU | 368 | 24 | yes | yes | yes | 3 | 65 | 70 | 20 | 41 | 42 |
| Hanania 2003 | FLU | SAL | 360 | 24 | yes | yes | yes | 3 | 65 | 70 | 20 | 41 | 42 |
| Hanania 2003 | FLU | FLU+ | 361 | 24 | yes | yes | yes | 3 | 65 | 70 | 20 | 41 | 42 |
| Hanania 2003 | PBO | SAL | 362 | 24 | yes | yes | yes | 3 | 65 | 70 | 20 | 41 | 42 |
| Hanania 2003 | FLU+ | SAL | 355 | 24 | yes | yes | yes | 3 | 65 | 70 | 20 | 41 | 42 |
| Hanania 2003 | PBO | FLU+ | 363 | 24 | yes | yes | yes | 3 | 65 | 70 | 20 | 41 | 42 |
| Szafranski 2003 | PBO | BUD | 403 | 52 | yes | yes | yes | 3 | 50 | 70 | 10 | 36 | 37 |
| Szafranski 2003 | BUD | FOR | 399 | 52 | yes | yes | yes | 3 | 50 | 70 | 10 | 36 | 37 |
| Szafranski 2003 | BUD | BUD+ | 406 | 52 | yes | yes | yes | 3 | 50 | 70 | 10 | 36 | 37 |
| Szafranski 2003 | PBO | FOR | 406 | 52 | yes | yes | yes | 3 | 50 | 70 | 10 | 36 | 37 |
| Szafranski 2003 | BUD+ | FOR | 409 | 52 | yes | yes | yes | 3 | 50 | 70 | 10 | 36 | 37 |
| Szafranski 2003 | PBO | BUD+ | 413 | 52 | yes | yes | yes | 3 | 50 | 70 | 10 | 36 | 37 |
| O Donnel 2004 | PBO | TIO | 187 | 6 | yes | yes | yes | 3 | 65 | NA | 10 | 41 | 42 |
| Briggs 2005 | SAL | TIO | 653 | 12 | yes | yes | yes | 3 | 60 | 70 | 10 | 37,7 | 37,7 |
| Campbell 2005 | PBO | FOR | 432 | 24 | yes | yes | yes | 4 | 70 | 70 | 10 | 53 | 54,4 |
| Niewoehner 2005 | PBO | TIO | 1829 | 24 | yes | yes | yes | 5 | 60 | 70 | 10 | 35,6 | 35,6 |
| van Noord 2005 | FOR | TIO | 139 | 18 | yes | yes | yes | 4 | 60 | 70 | 10 | 37,2 | 37,2 |
| Barnes 2006 | PBO | FLU+ | 140 | 13 | yes | yes | yes | 3 | NA | NA | NA | 58 | 59 |
| Beeh 2006 | PBO | TIO | 1639 | 12 | yes | yes | yes | 3 | 70 | 70 | 10 | 45,3 | 45,7 |
| Dusser 2006 | PBO | TIO | 1010 | 48 | yes | yes | NA | 2 | 65 | 70 | 10 | 47,6 | 48,2 |
| O Donnel 2006 | PBO | FLU+ | 126 | 8 | yes | yes | yes | 3 | 70 | 70 | 10 | 39,5 | 42,5 |
| O Donnel 2006 | FLU+ | SAL | 121 | 8 | yes | yes | yes | 3 | 70 | 70 | 10 | 39,5 | 42,5 |
| O Donnel 2006 | PBO | SAL | 123 | 8 | yes | yes | yes | 3 | 70 | 70 | 10 | 39,5 | 42,5 |
| Stokley 2006 | PBO | SAL | 634 | 52 | yes | yes | yes | 5 | 70 | NA | NA | 46,1 | 45,8 |
| Verkindre 2006 | PBO | TIO | 87 | 12 | yes | yes | no | 2 | 50 | 70 | 10 | 34,7 | 35,8 |
| Baumgartner 2007 | PBO | SAL | 287 | 12 | yes | yes | yes | 4 | 65 | 70 | 15 | 40,6 | 41,6 |
| Freeman 2007 | PBO | TIO | 395 | 12 | yes | yes | yes | 4 | 65 | 70 | 10 | 47,9 | 49,9 |
| Calverley 2007 | PBO | FLU | 3058 | 156 | yes | yes | yes | 4 | 60 | 70 | 10 | 43,6 | 44,3 |
| Calverley 2007 | FLU | SAL | 3055 | 156 | yes | yes | yes | 4 | 60 | 70 | 10 | 43,6 | 44,3 |
| Calverley 2007 | FLU | FLU+ | 3067 | 156 | yes | yes | yes | 4 | 60 | 70 | 10 | 43,6 | 44,3 |
| Calverley 2007 | PBO | SAL | 3045 | 156 | yes | yes | yes | 4 | 60 | 70 | 10 | 43,6 | 44,3 |
| Calverley 2007 | FLU+ | SAL | 3054 | 156 | yes | yes | yes | 4 | 60 | 70 | 10 | 43,6 | 44,3 |
| Calverley 2007 | PBO | FLU+ | 3057 | 156 | yes | yes | yes | 4 | 60 | 70 | 10 | 43,6 | 44,3 |
| Kardos 2007 | FLU+ | SAL | 994 | 44 | yes | yes | yes | 4 | 50 | 70 | 10 | 40,3 | 40,4 |
| Powrie 2007 | PBO | TIO | 142 | 52 | yes | yes | yes | 3 | 80 | 70 | 10 | 49,2 | 50,9 |
| Wedzicha 2008 | FLU+ | TIO | 1323 | 104 | yes | yes | yes | 4 | 50 | NA | 10 | 39,1 | 39,4 |
| Dal Negro 2003 | PBO | FLU+ | 12 | 52 | yes | yes | yes | 3 | 80 | 70 | 10 | 48 | 50 |
| Dal Negro 2003 | FLU+ | SAL | 12 | 52 | yes | yes | yes | 3 | 80 | 70 | 10 | 48 | 50 |
| Dal Negro 2003 | PBO | SAL | 12 | 52 | yes | yes | yes | 3 | 80 | 70 | 10 | 48 | 50 |

BUD, budesonide; BUD+, budesonide plus formoterol; Descr withdraw, description of withdrawals; FEV_1_, forced expiratory volume in 1 second; FLU, fluticasone; FLU+, fluticasone plus salmeterol; FOR, formoterol; FVC, forced vital capacity; NA, not available; SAL, salmeterol; TIO, tiotropium; PBO, placebo; Random alloc, random allocation.

**Table S3. Linkage methods used**

| **Linkage method** | **Explanation** |
| --- | --- |
| average | The *average* of the dissimilarities between all elements of two clusters. |
| centroid | The dissimilarity between the *centroids* of the two clusters. It may result in inversions [1]. |
| complete | The *maximum* of the dissimilarities between all elements of two clusters. |
| McQuitty's | The *average* of the distances between each element of two clusters. |
| median | The *median* of the dissimilarities between all elements of two clusters. |
| single | The *minimum* of the dissimilarities between all elements of two clusters. It may result in a chaining effect, creating strand-like clusters [1]. |
| Ward's D | The *largest ratio* of the intercluster sum of *absolute* distances to the total sum of *absolute* distances. |
| Ward's D2 | The *largest ratio* of the intercluster sum of *squared* distances to the total sum of *squared* distances. It is equivalent to the coefficient of determination in regression. |

**References**

1. James G, Witten D, Hastie T, Tibshirani R. An Introduction to Statistical Learning: with Applications in R. 2nd Edition, New York (NY): Springer; 2021.

**Table S4. Optimal linkage method (rheumatoid arthritis)**

| **Linkage methods** | **CCC value** |
| --- | --- |
| average | 0.881 |
| McQuitty | 0.878 |
| complete | 0.876 |
| Ward's D2 | 0.872 |
| centroid | 0.872 |
| Ward's D | 0.867 |
| single | 0.865 |
| median | 0.856 |

CCC, cophenetic correlation coefficient.

**Table S5. Optimal linkage method (COPD)**

| **Linkage methods** | **CCC value** |
| --- | --- |
| average | 0.822 |
| centroid | 0.820 |
| McQuitty | 0.803 |
| single | 0.749 |
| median | 0.728 |
| Ward's D2 | 0.669 |
| Ward's D | 0.606 |
| complete | 0.600 |

CCC, Cophenetic correlation coefficient.

**Supplementary Figures**


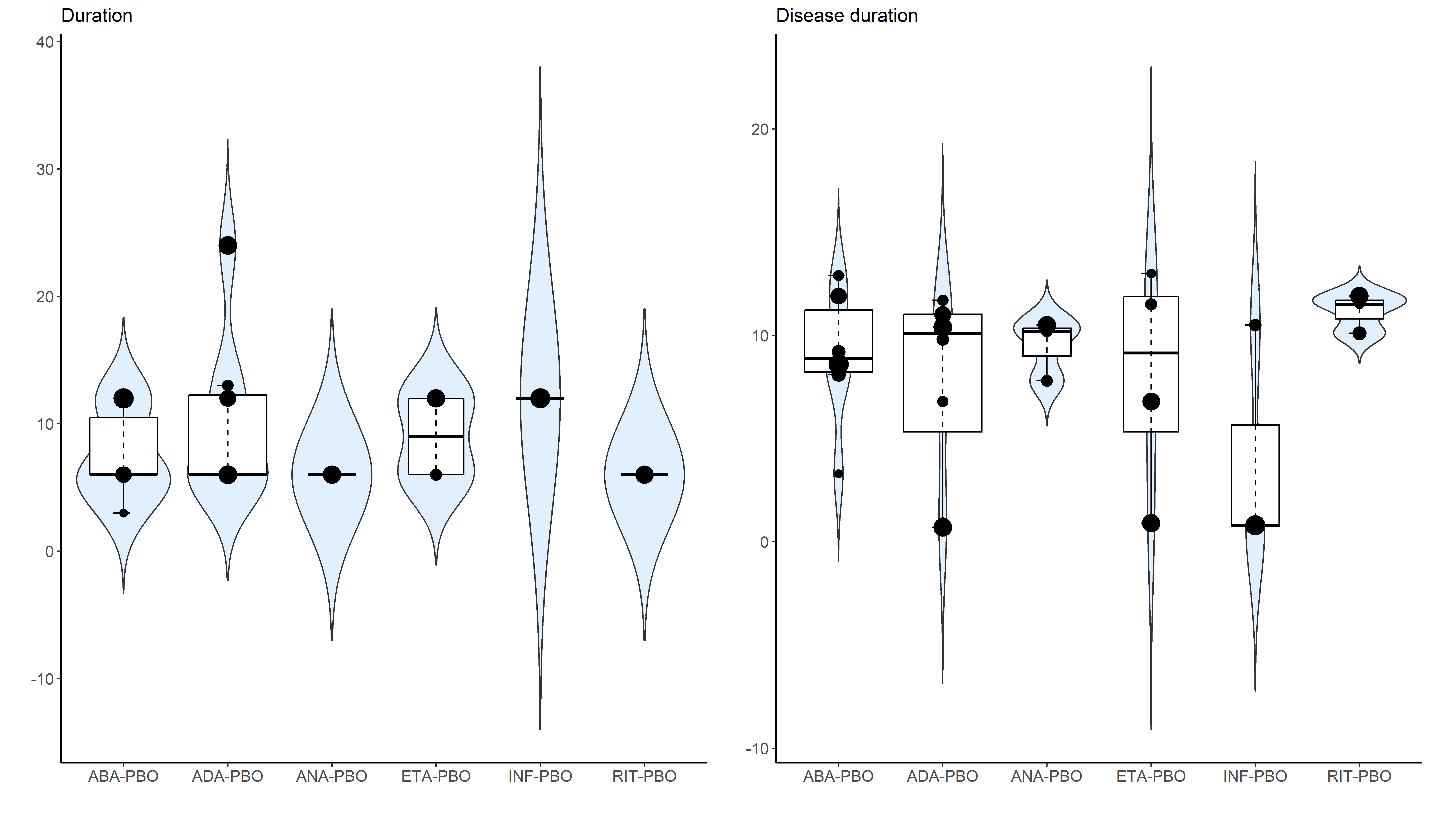


**Figure S1.** Violin plots (with integrated box plots and dots) for two quantitative characteristics extracted from the Cochrane review on rheumatoid arthritis.^14^ Each dot corresponds to a study conducted for the corresponding comparison on the x-axis. The size of the dots is proportional to the total sample size of the studies: larger dots correspond to larger studies.

ABA, abatacept; ADA, adalimumab; ANA, anakinra; ETA, etanercept; INF, infliximab; PBO, placebo; RIT, rituximab.


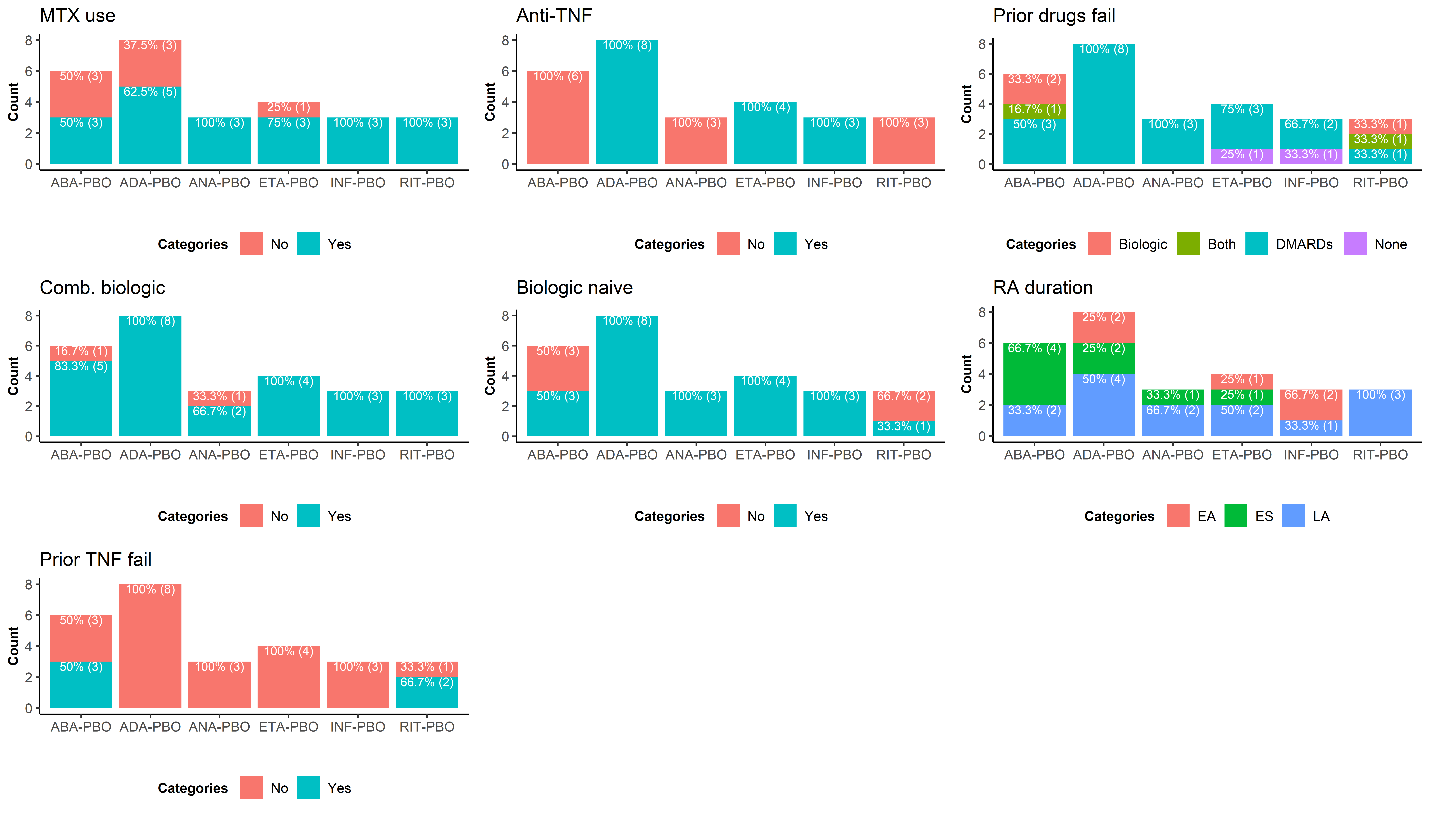


**Figure S2.** Stacked bar plots for several qualitative characteristics extracted from the Cochrane review on rheumatoid arthritis.^14^ The relative and absolute frequencies (in parenthesis) refer to each comparison on the x-axis.

ABA, abatacept; ADA, adalimumab; ANA, anakinra; Comb. biologic, combination biologic therapy; DMARDs, disease-modifying antirheumatic drug; ETA, etanercept; INF, infliximab; MTX use, concomitant use of MTX; PBO, placebo; RA, rheumatoid arthritis; RIT, rituximab; TNF, tumour necrosis factor.


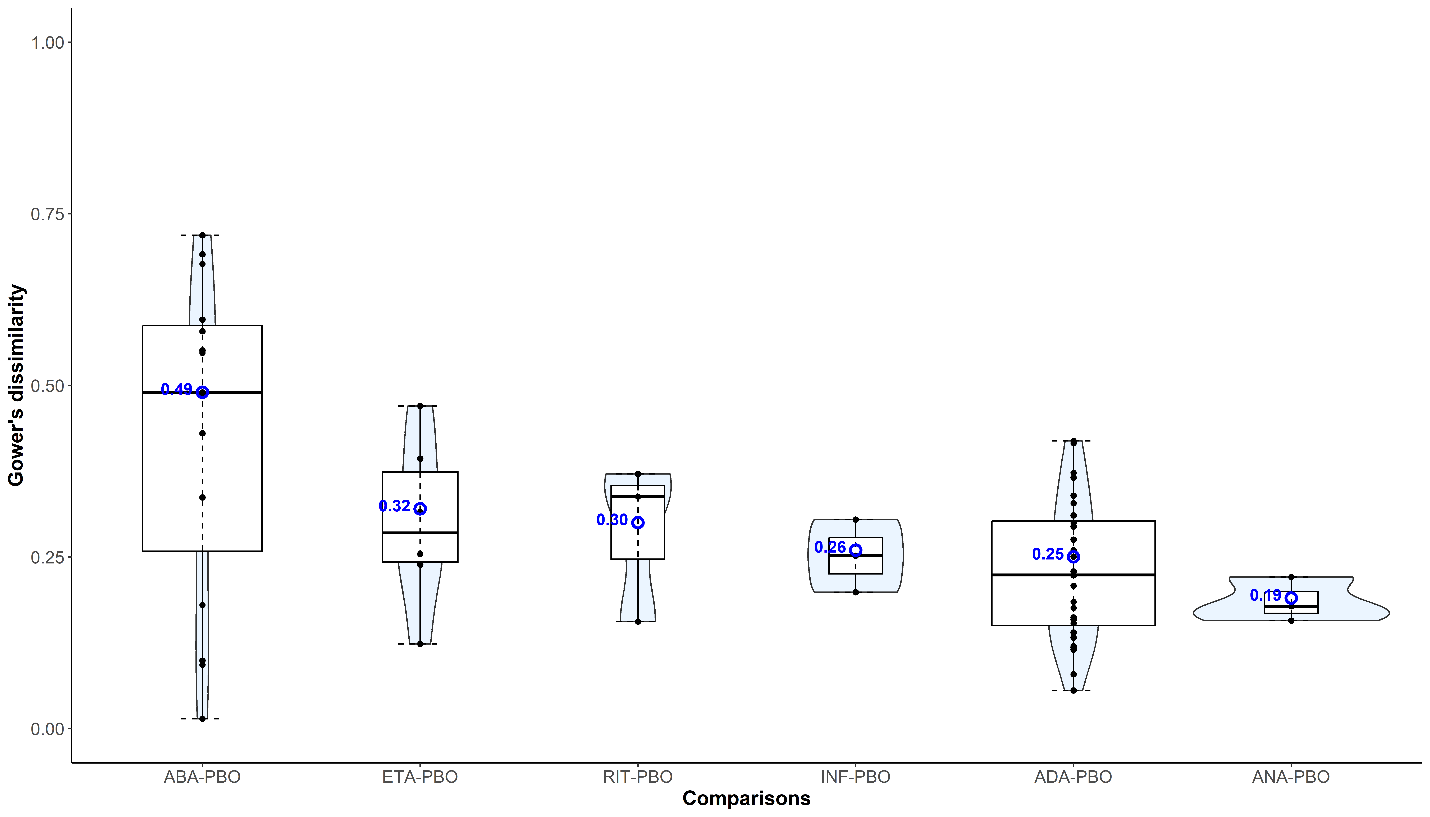


**Figure S3.** Violin plots with integrated box plots and dots on Gower's dissimilarity coefficient of study pairs (black dots) and within-comparison dissimilarity (blue dots) for each comparison. The black points stem from the off-diagonal elements of the $\left\{ d \right\}_{27\times27}$ dissimilarity matrix. The violins have been sorted in decreasing order of the within-comparison dissimilarity. Analysis was performed on the network for rheumatoid arthritis.^14^ ABA, abatacept; ADA, adalimumab; ANA, anakinra; ETA, etanercept; INF, infliximab; PBO, placebo; RIT, rituximab


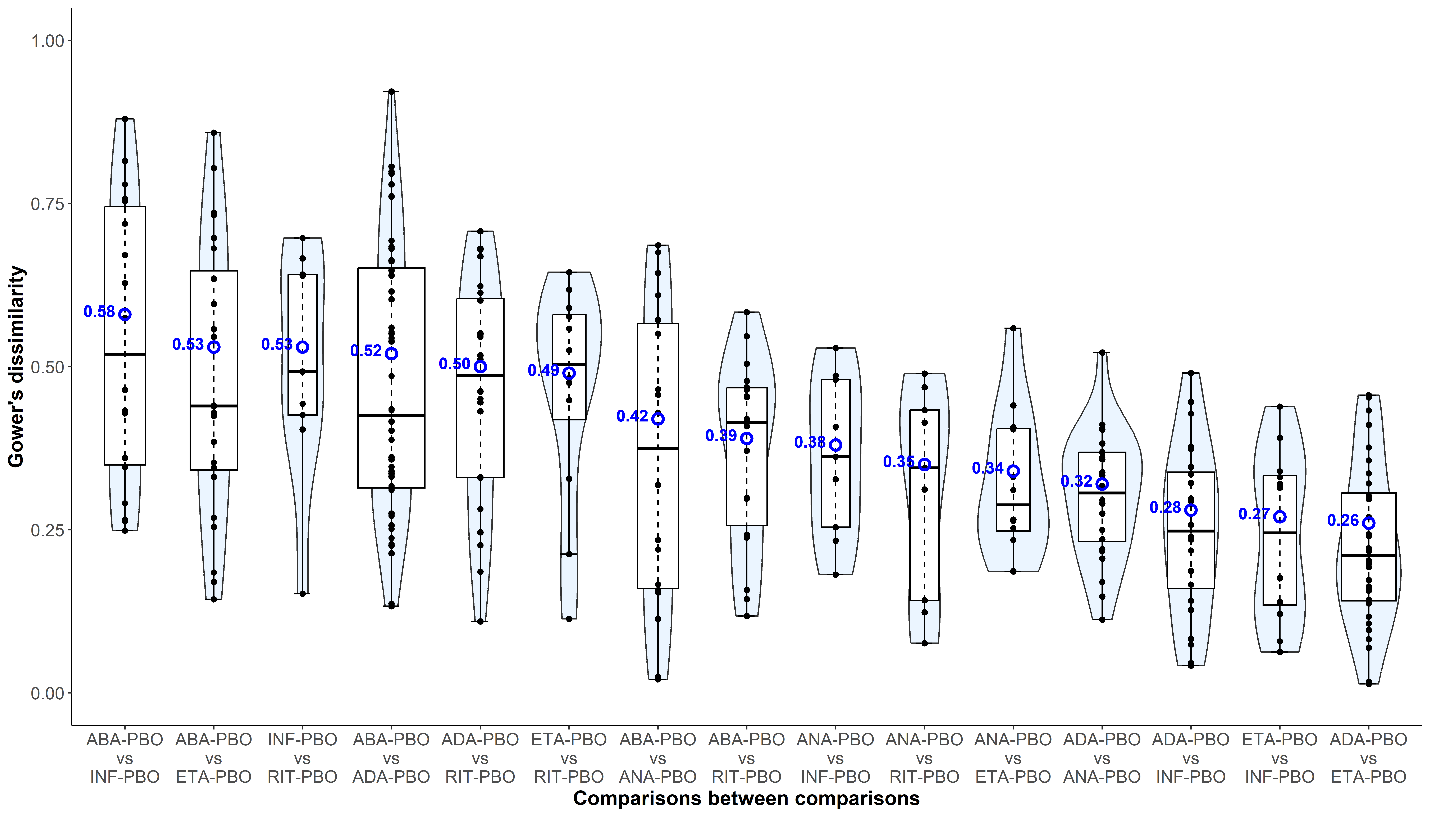


**Figure S4.** Violin plots with integrated box plots and dots on Gower's dissimilarity coefficient of study pairs (black dots) and between-comparison dissimilarity (blue dots) for each comparison between comparisons. The black points stem from the off-diagonal elements of the $\left\{ d \right\}_{27\times27}$ dissimilarity matrix. The violins have been sorted in decreasing order of the between-comparison dissimilarity. Analysis was performed on the network for rheumatoid arthritis.^14^ ABA, abatacept; ADA, adalimumab; ANA, anakinra; ETA, etanercept; INF, infliximab; PBO, placebo; RIT, rituximab


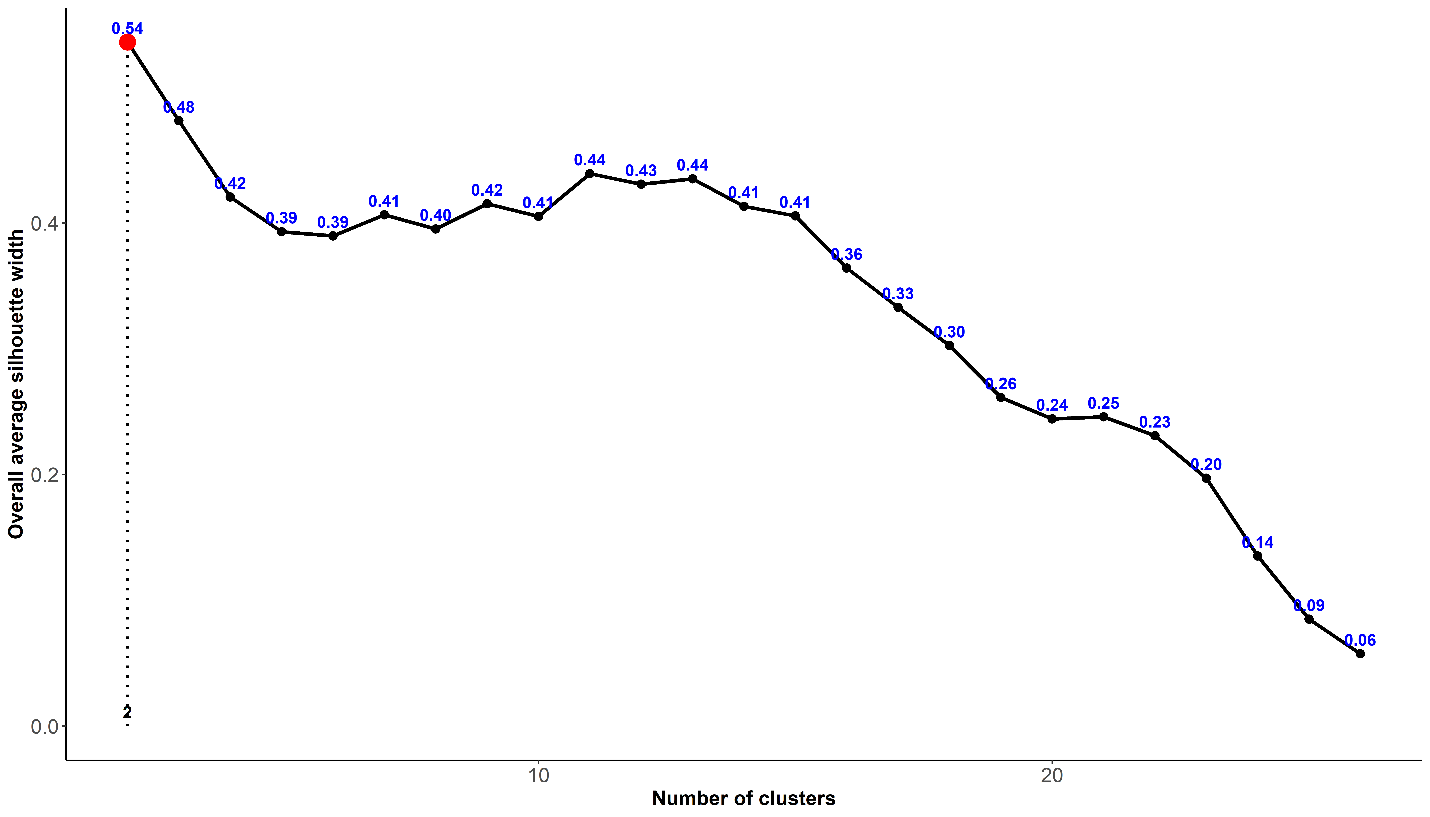


**Figure S5.** Profile plot for overall average silhouette width to detect the optimal partition for the comparisons in the network for rheumatoid arthritis.^14^ The x-axis refers to the investigated range of clusters (from two to $\sum_{i=1}^{27} \binom{\left| T_{i} \right|}{2}-1=27-1$, with $\left| T_{i} \right|$ being the number of treatments in the study $i$), and the y-axis refers to the overall average silhouette width for the corresponding investigated cluster. The red point indicates the optimal partition corresponding to the largest overall average silhouette width.


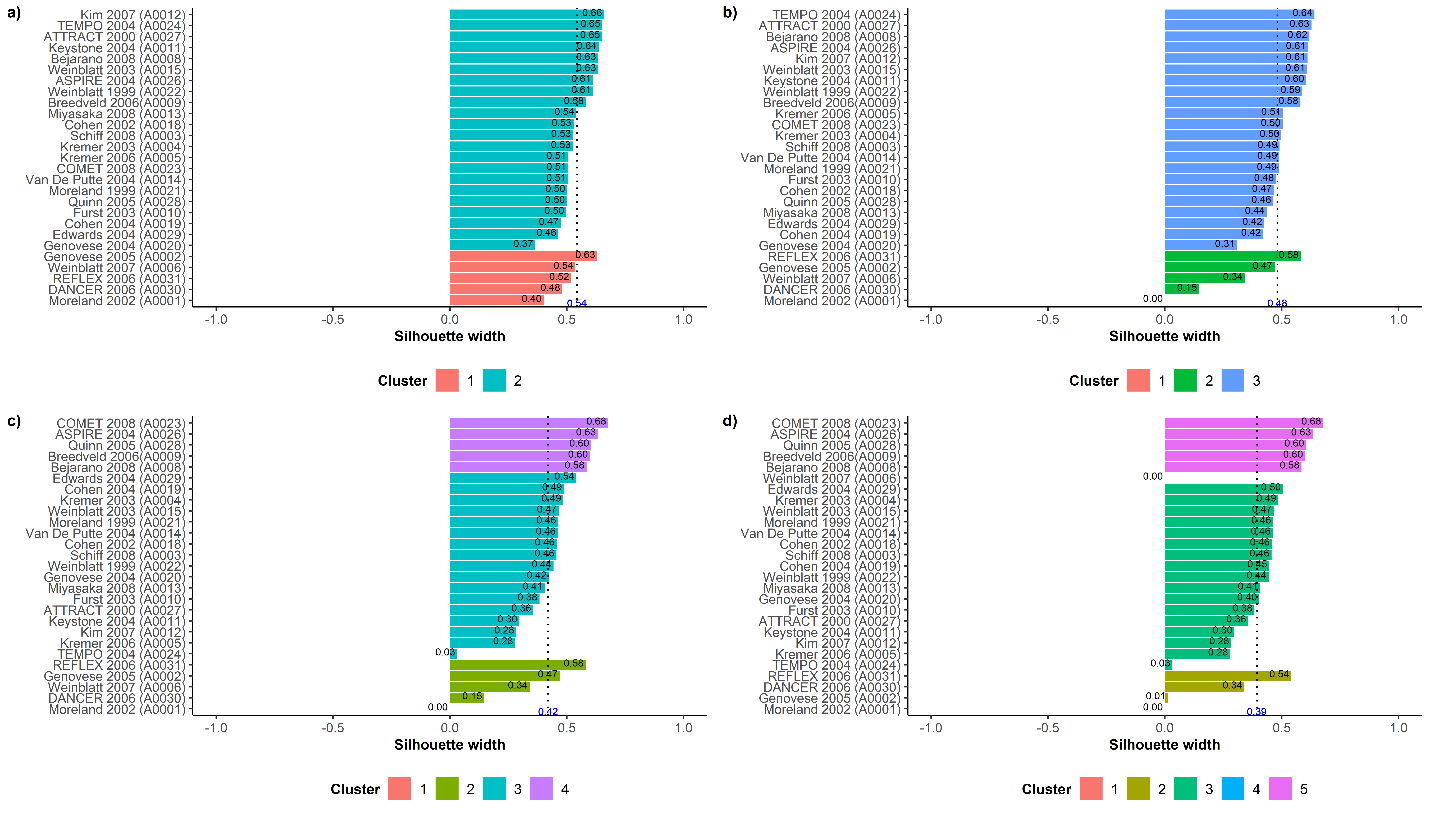


**Figure S6.** Silhouette width plots for different numbers of clusters for the network for rheumatoid arthritis^14^: two clusters (plot a)), three clusters (plot b)), four clusters (plot c)) and five clusters (plot d)). Each bar refers to the silhouette width (x-axis) of the corresponding study (y-axis). The bars are sorted in decreasing order of the silhouette width within each cluster. The silhouette width value appears on each bar. Different colours refer to the different clusters. The vertical dotted line refers to the overall average silhouette width (number in blue). Note that the order of the studies in the y-axis differs across the plots.

Note that the third cluster in the three-cluster solution (plot b) highlights a group of studies that may warrant a separate cluster (Bejarano 2008, Breedveld 2006, COMET 2008, ASPIRE 2004, and Quinn 2005). These studies share several distinct characteristics: all have disease durations of less than one year, compared to at least 3.3 years in the other clusters; their study durations are all at least one year, whereas other studies have durations of at most one year; and six out of seven categorical variables align perfectly. The optimal partitioning as dictated by the average Silhouette width may not always be 'optimal', and the analysts should consider the first two or three optimal partitions when inspecting the characteristics' distribution by cluster.


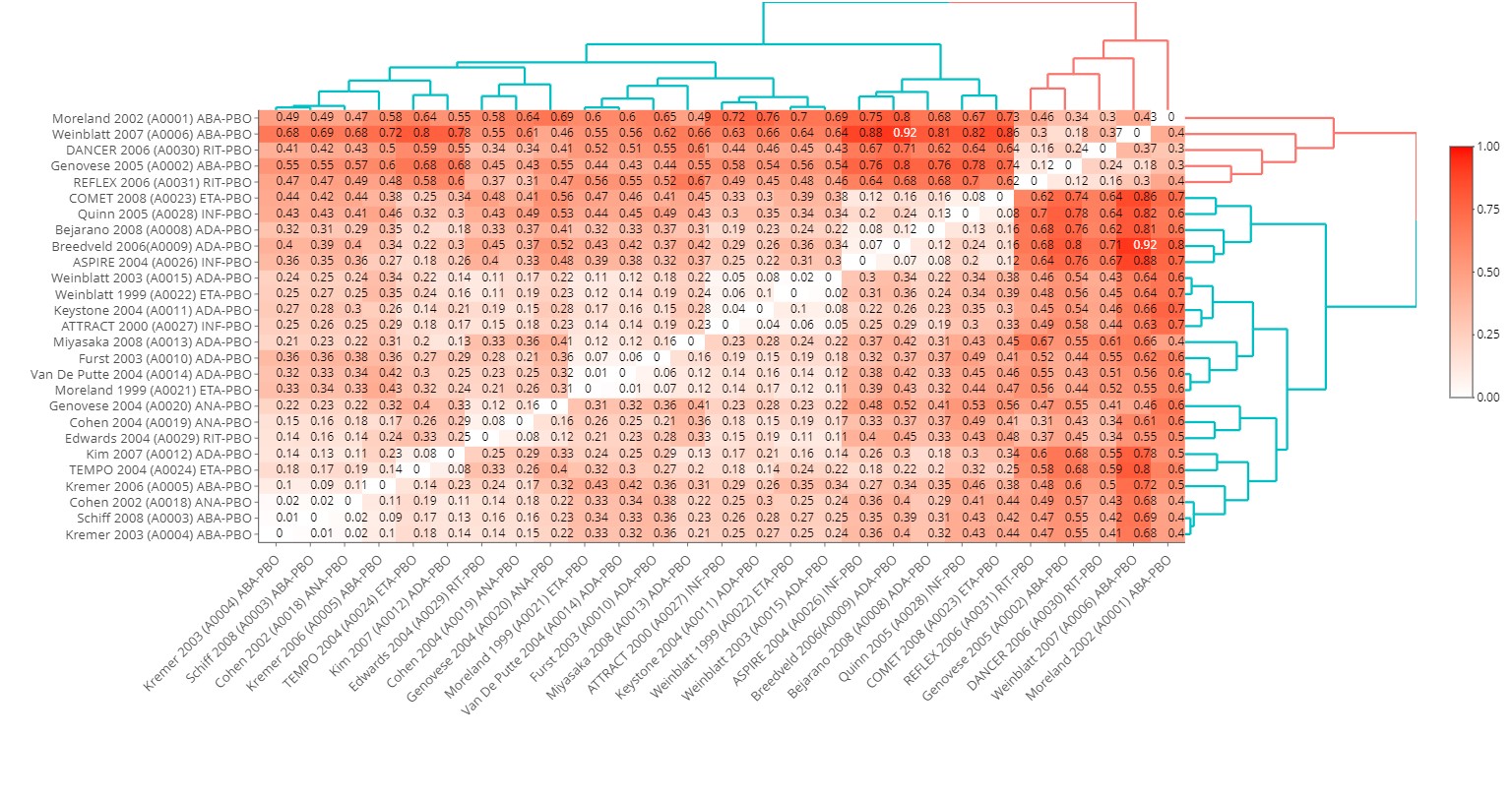
**Figure S7.** A heatmap on Gower's dissimilarity among the studies in the network and integrated dendrogram of two clusters. Darker shades of red refer to a higher dissimilarity between the compared studies. The leaves refer to the study name and corresponding treatment comparison. The clusters are indicated with different colours of the branches. Analysis was performed on the network for rheumatoid arthritis.^14^

ABA, abatacept; ADA, adalimumab; ANA, anakinra; ETA, etanercept; INF, infliximab; PBO, placebo; RIT, rituximab.


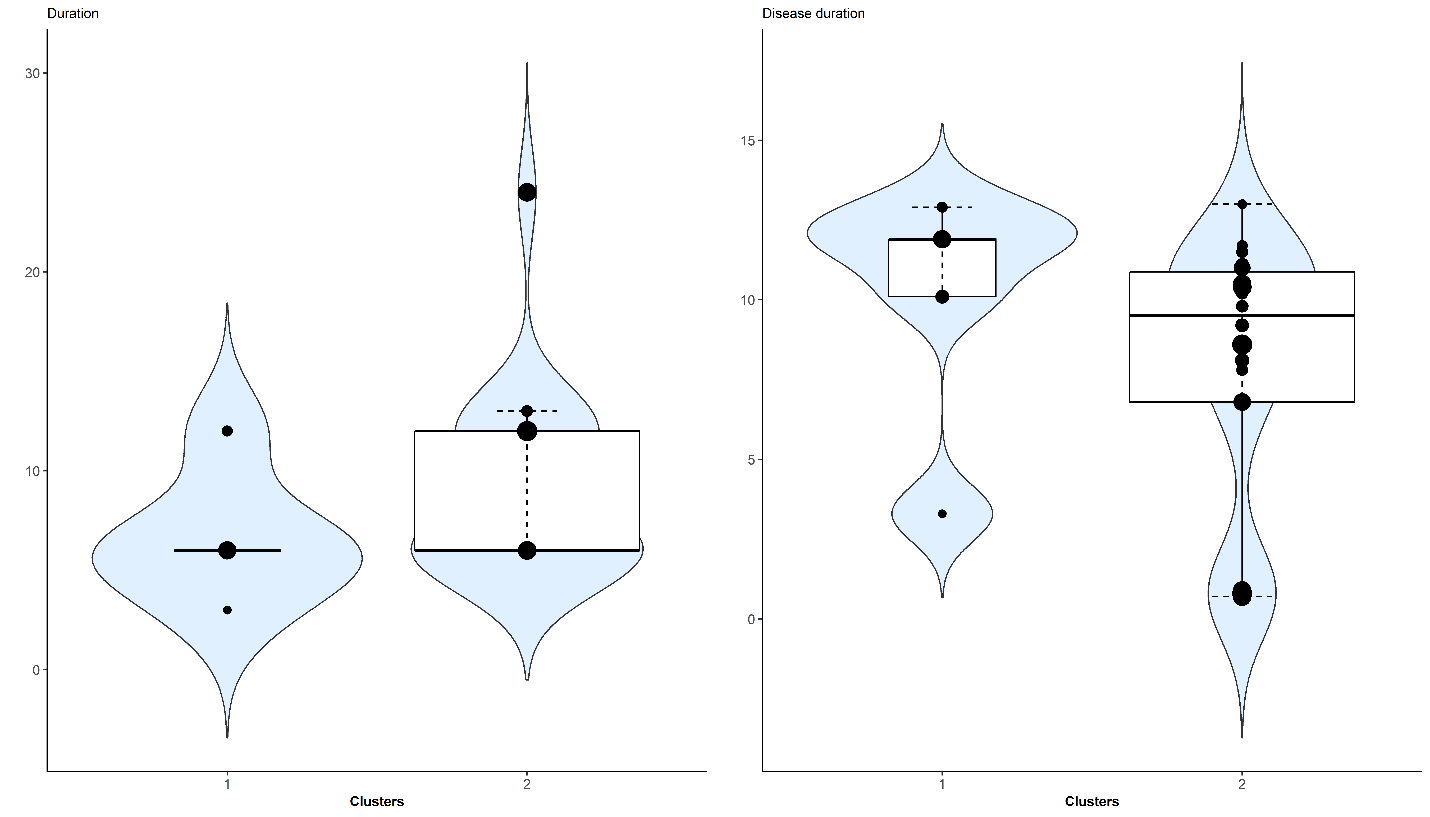


**Figure S8.** Violin plots (with integrated box plots and dots) for two quantitative characteristics extracted from the Cochrane review on rheumatoid arthritis.^14^ Each dot corresponds to a study in the corresponding cluster (x-axis), with the size of the dots being proportional to the total sample size of the studies: larger dots correspond to larger studies.


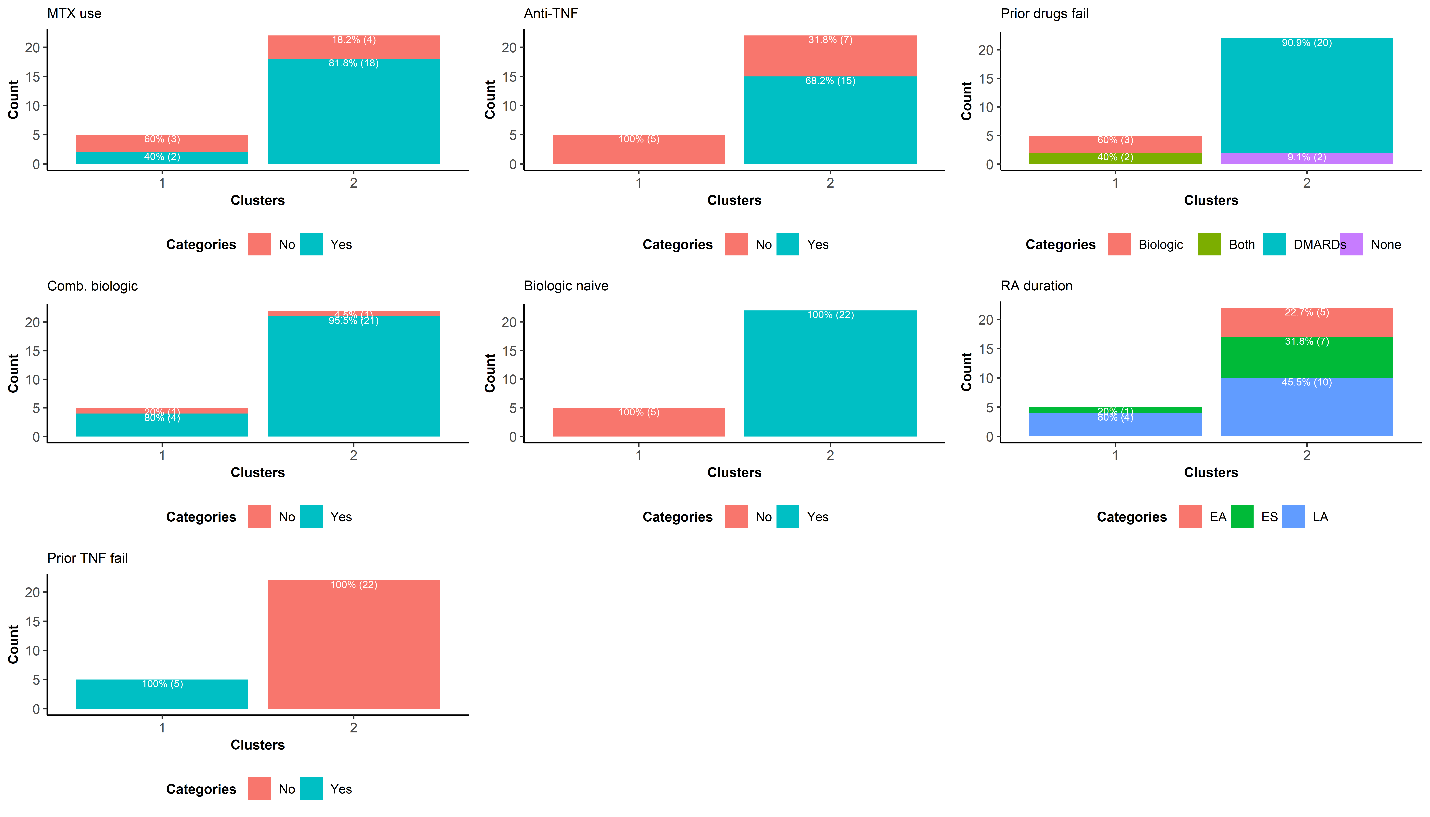


**Figure S9.** Stacked bar plots for several qualitative characteristics extracted from the Cochrane review on rheumatoid arthritis.^14^ The relative and absolute frequencies (in parenthesis) refer to each cluster on the x-axis.

Comb. biologic, combination biologic therapy; DMARDs, disease-modifying antirheumatic drug; MTX use, concomitant use of MTX; RA, rheumatoid arthritis; TNF, tumour necrosis factor.


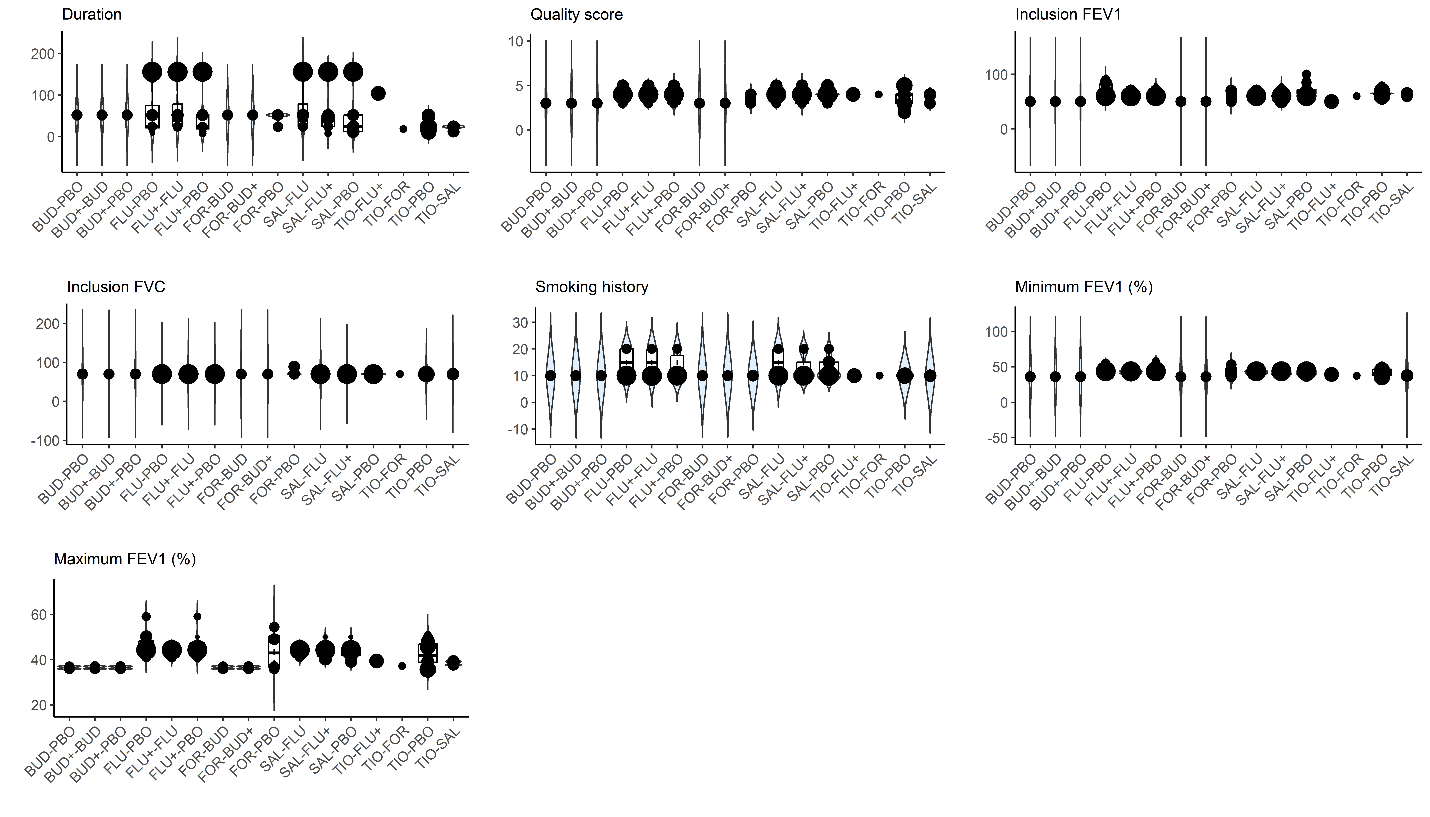


**Figure S10.** Violin plots (with integrated box plots and dots) for several quantitative characteristics extracted from the systematic review on chronic obstructive pulmonary disease.^15^ Each dot corresponds to a study conducted for the corresponding comparison on the x-axis. The size of the dots is proportional to the total sample size of the studies: larger dots correspond to larger studies.

BUD, budesonide; BUD+, budesonide plus formoterol; FEV1, forced expiratory volume in 1 second; FLU, fluticasone; FLU+, fluticasone plus salmeterol; FOR, formoterol; FVC, forced vital capacity; PBO. placebo; SAL, salmeterol; TIO, tiotropium.


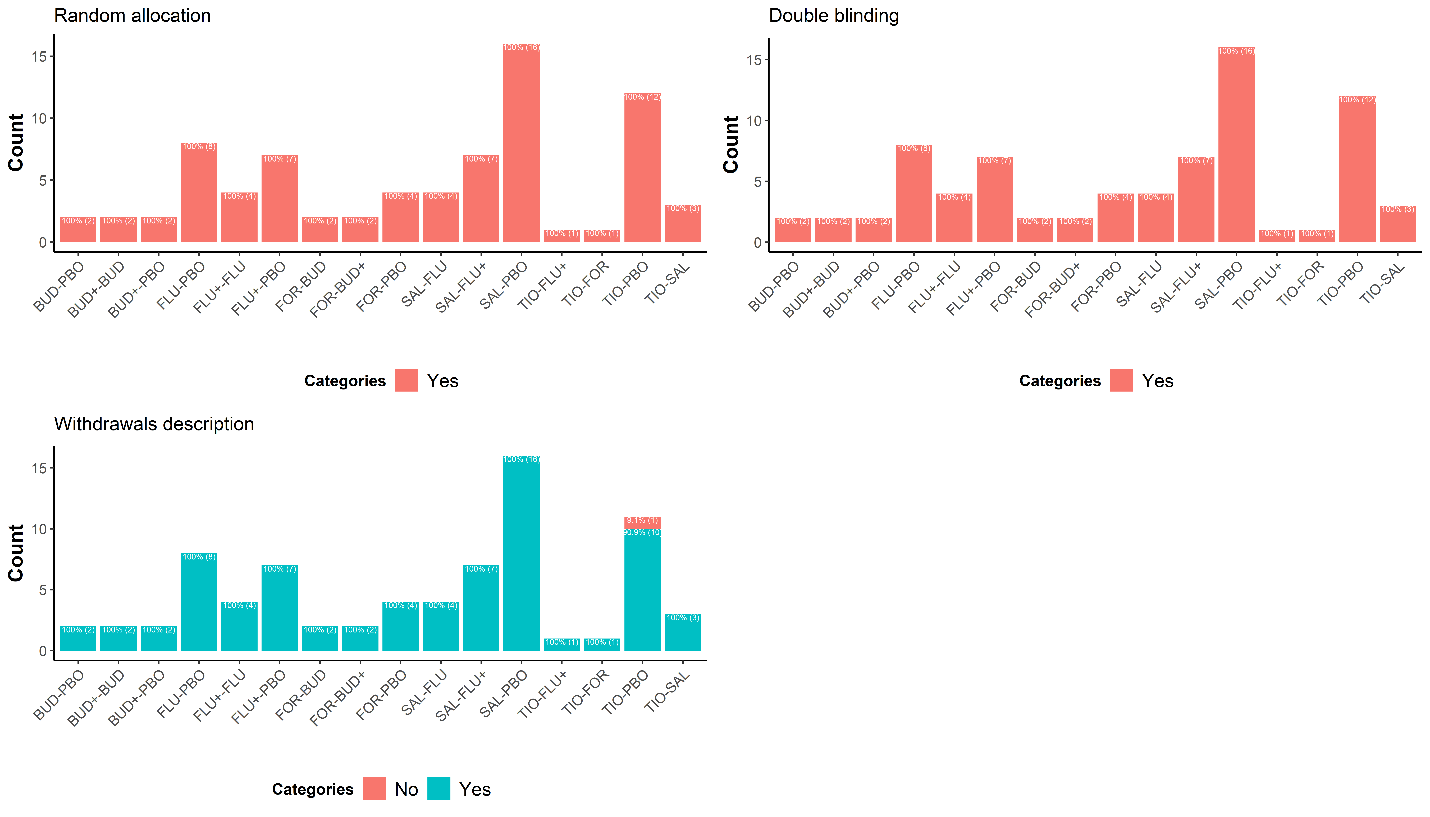


**Figure S11.** Stacked bar plots for several qualitative characteristics extracted from the systematic review on chronic obstructive pulmonary disease.^15^ The relative and absolute frequencies (in parenthesis) refer to each comparison on the x-axis.

BUD, budesonide; BUD+, budesonide plus formoterol; FLU, fluticasone; FLU+, fluticasone plus salmeterol; FOR, formoterol; PBO, placebo; SAL, salmeterol; TIO, tiotropium.


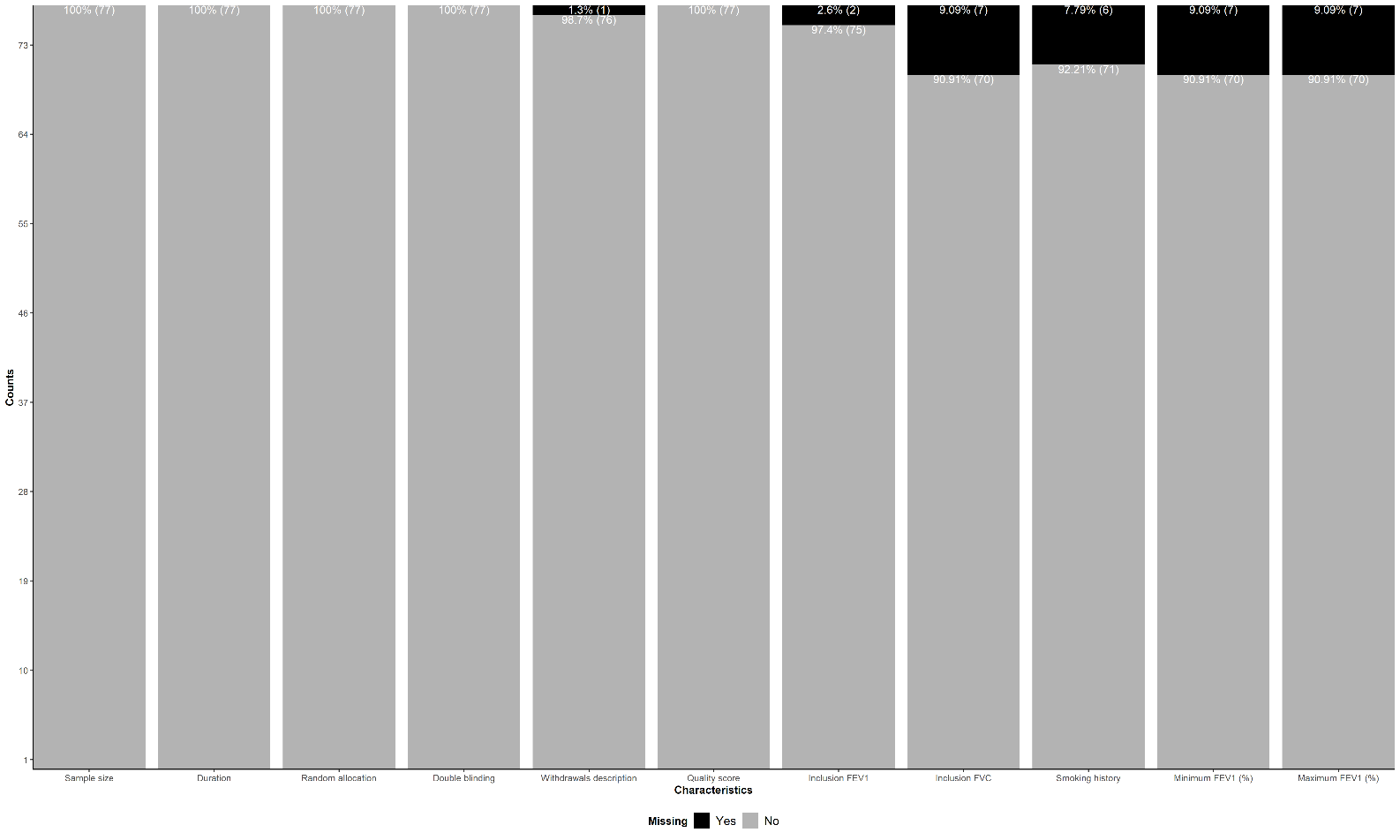
**Figure S12.** A stacked bar plot for the amount of missing and observed data for each characteristic from the systematic review on chronic obstructive pulmonary disease.^15^ Black and grey bars refer to the relative and absolute (in parenthesis) number of missing and observed data for each characteristic.

FEV1, forced expiratory volume in 1 second; FVC, forced vital capacity.


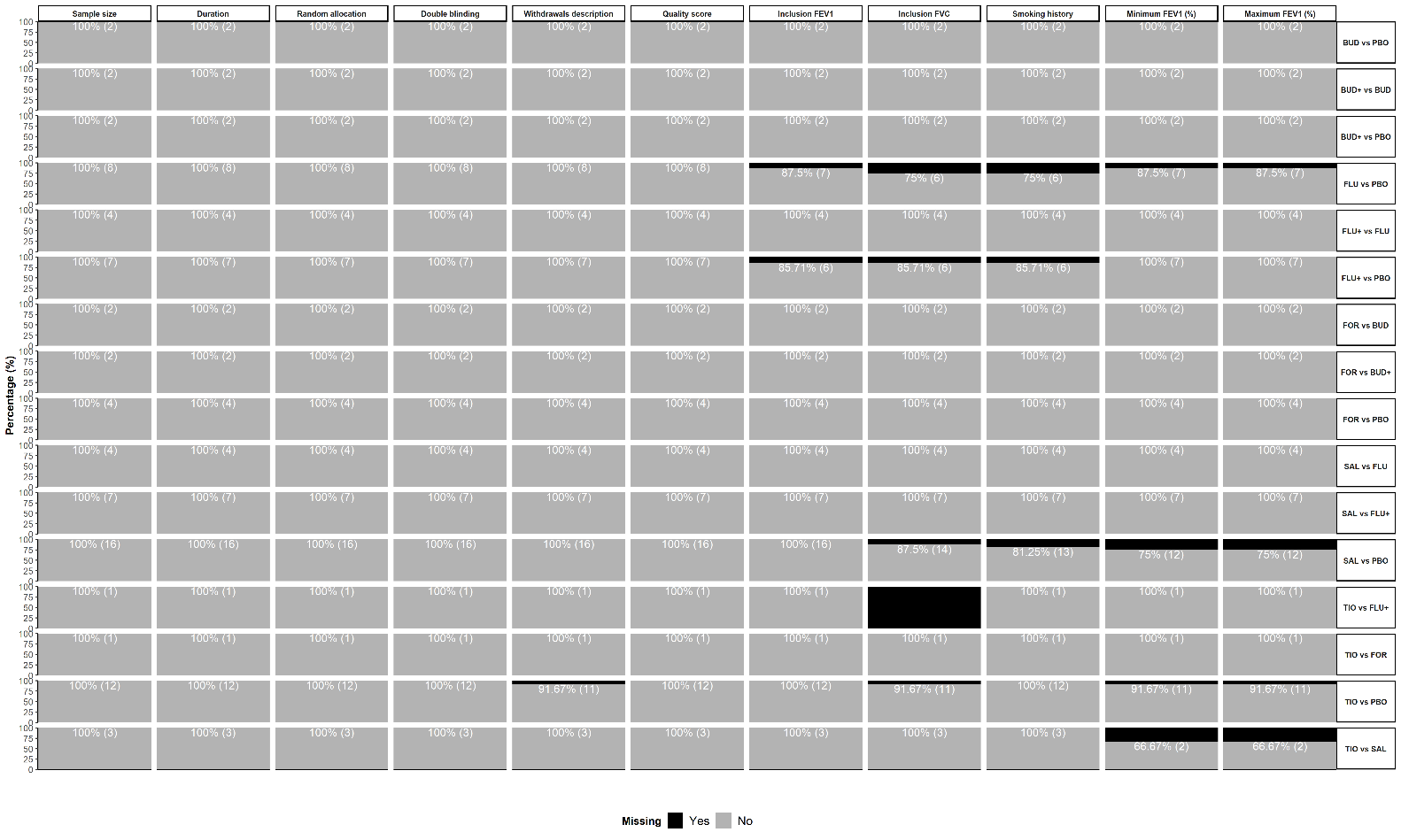
**Figure S13.** Stacked bars for the amount of missing and observed data for each combination of characteristics with comparison. Characteristics extracted from the systematic review on obstructive pulmonary disease.^15^ Black and grey bars refer to missing and observed data for each characteristic. The relative and absolute (in parenthesis) frequencies appear only for the observed data.

BUD, budesonide; BUD+, budesonide plus formoterol; FEV1, forced expiratory volume in 1 second; FLU, fluticasone; FLU+, fluticasone plus salmeterol; FOR, formoterol; FVC, forced vital capacity; PBO, placebo; SAL, salmeterol; TIO, tiotropium.


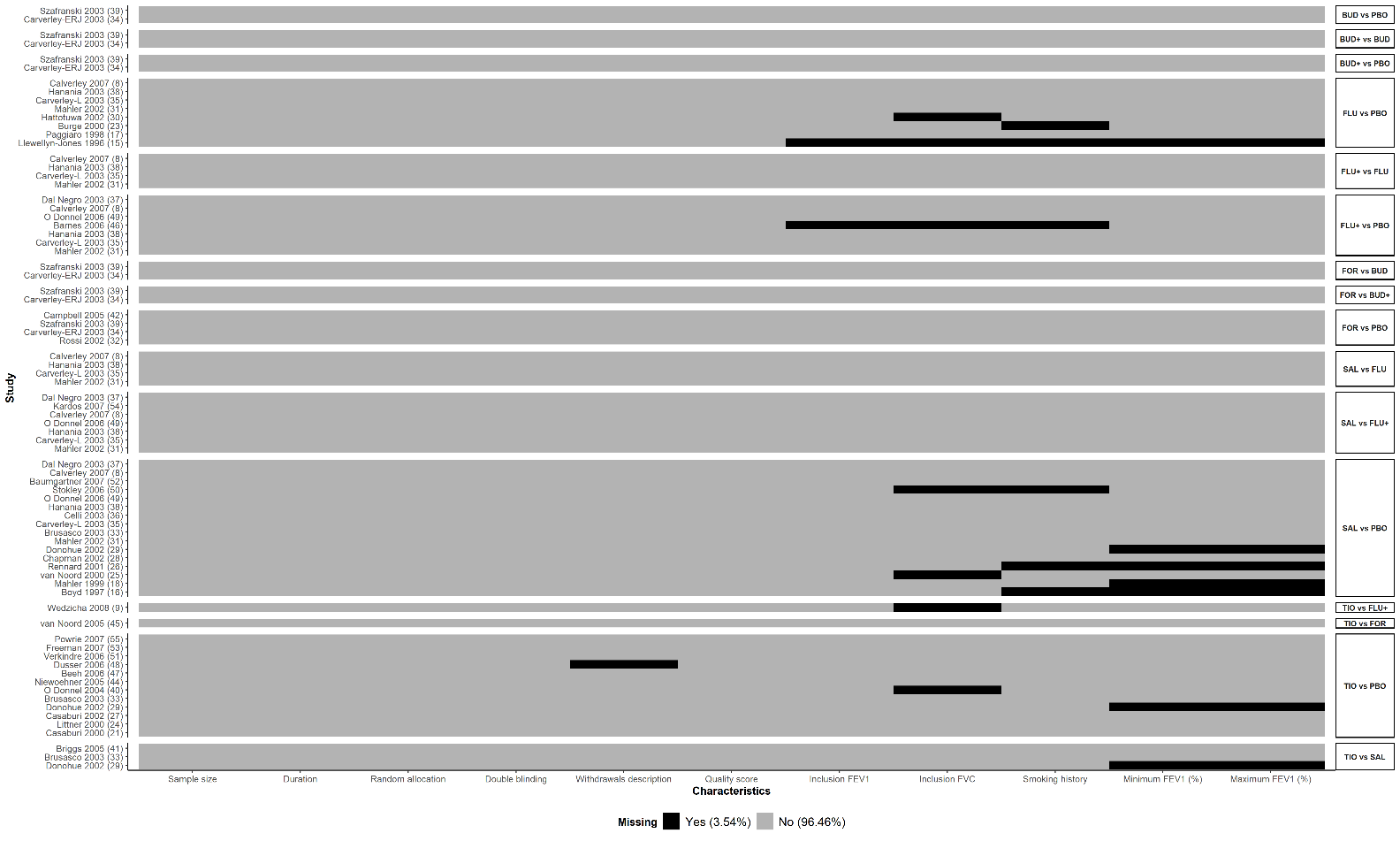
**Figure S14.** Tile plots on the pattern of missing data for each comparison from the systematic review on chronic obstructive pulmonary disease.^15^ The x-axis refers to the extracted characteristics, and the y-axis refers to the studies investigating the corresponding comparisons. Black tiles indicate that one or more characteristics were not reported in the corresponding studies.

BUD, budesonide; BUD+, budesonide plus formoterol; FEV1, forced expiratory volume in 1 second; FLU, fluticasone; FLU+, fluticasone plus salmeterol; FOR, formoterol; FVC, forced vital capacity; PBO, placebo; SAL, salmeterol; TIO, tiotropium.

**
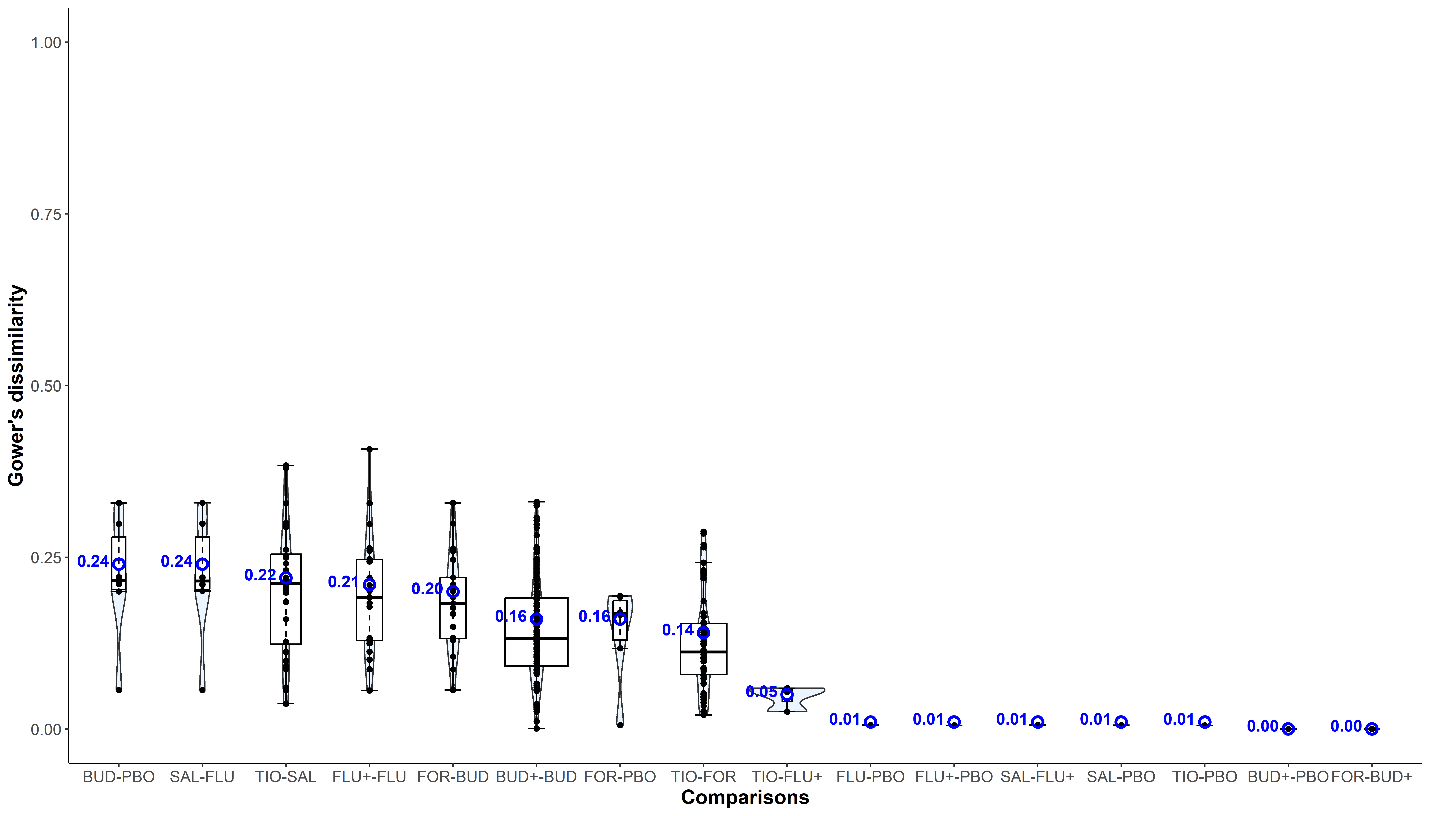
**

**Figure S15.** Violin plots with integrated box plots and dots on Gower's dissimilarity coefficient of study pairs (black dots) and within-comparison dissimilarity (red dots) for each comparison. The black points stem from the off-diagonal elements of the $\left\{ d \right\}_{77\times77}$ dissimilarity matrix. The violins have been sorted in decreasing order of the within-comparison dissimilarity. Analysis was performed on the network for chronic obstructive pulmonary disease.^15^

BUD, budesonide; BUD+, budesonide plus formoterol; FLU, fluticasone; FLU+, fluticasone plus salmeterol; FOR, formoterol; PBO, placebo; SAL, salmeterol; TIO, tiotropium.


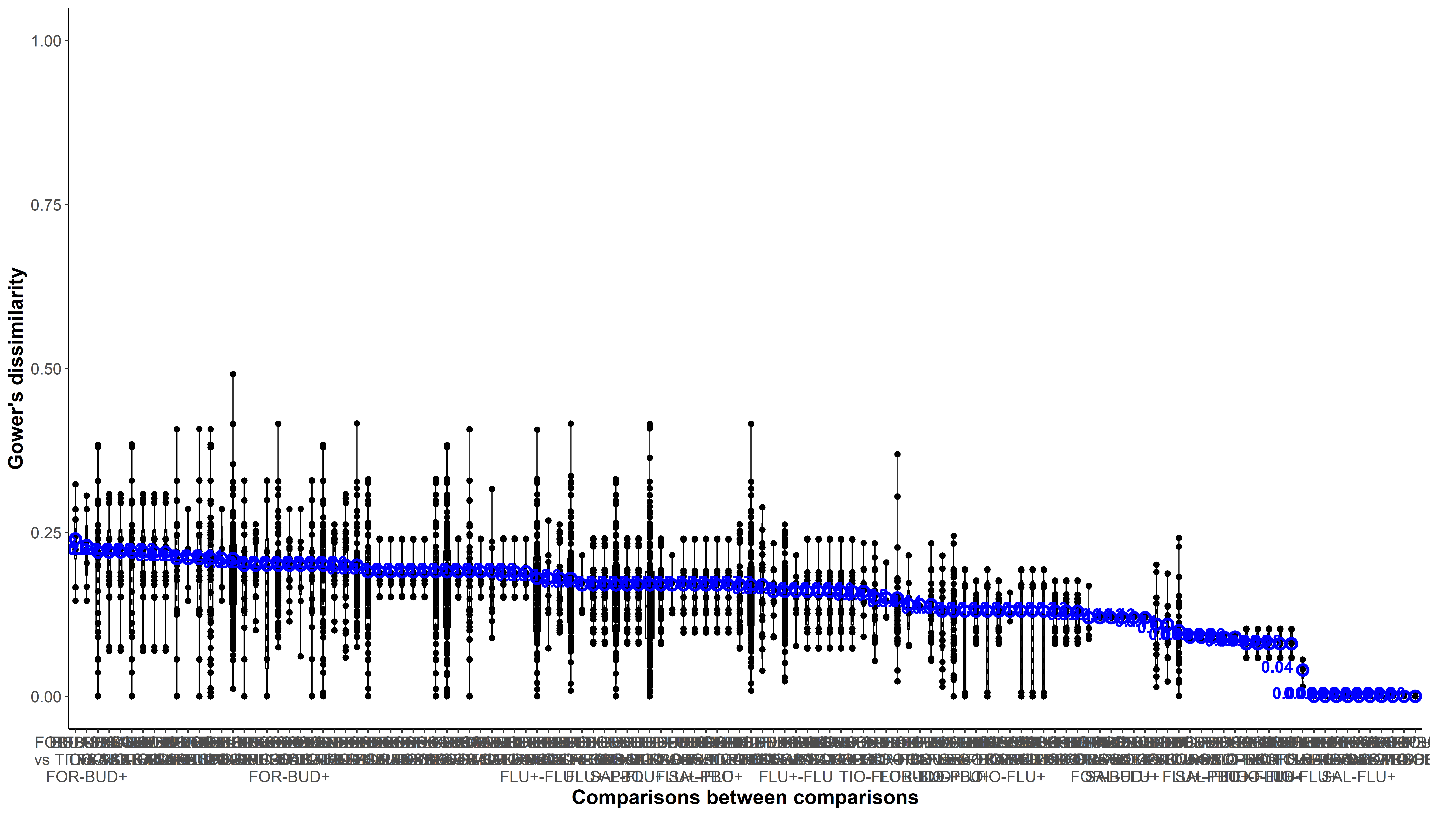
**Figure S16.** Violin plots with integrated box plots and dots on Gower's dissimilarity coefficient of study pairs (black dots) and between-comparison dissimilarity (blue dots) for each comparison. The black points stem from the off-diagonal elements of the $\left\{ d \right\}_{77\times77}$ dissimilarity matrix. The violins have been sorted in decreasing order of the between-comparison dissimilarity. Analysis was performed on the network for chronic obstructive pulmonary disease.^15^ The names of the comparisons between comparisons overlap with each other for stemming from a network with many observed comparisons, creating a 'cluttering' effect.


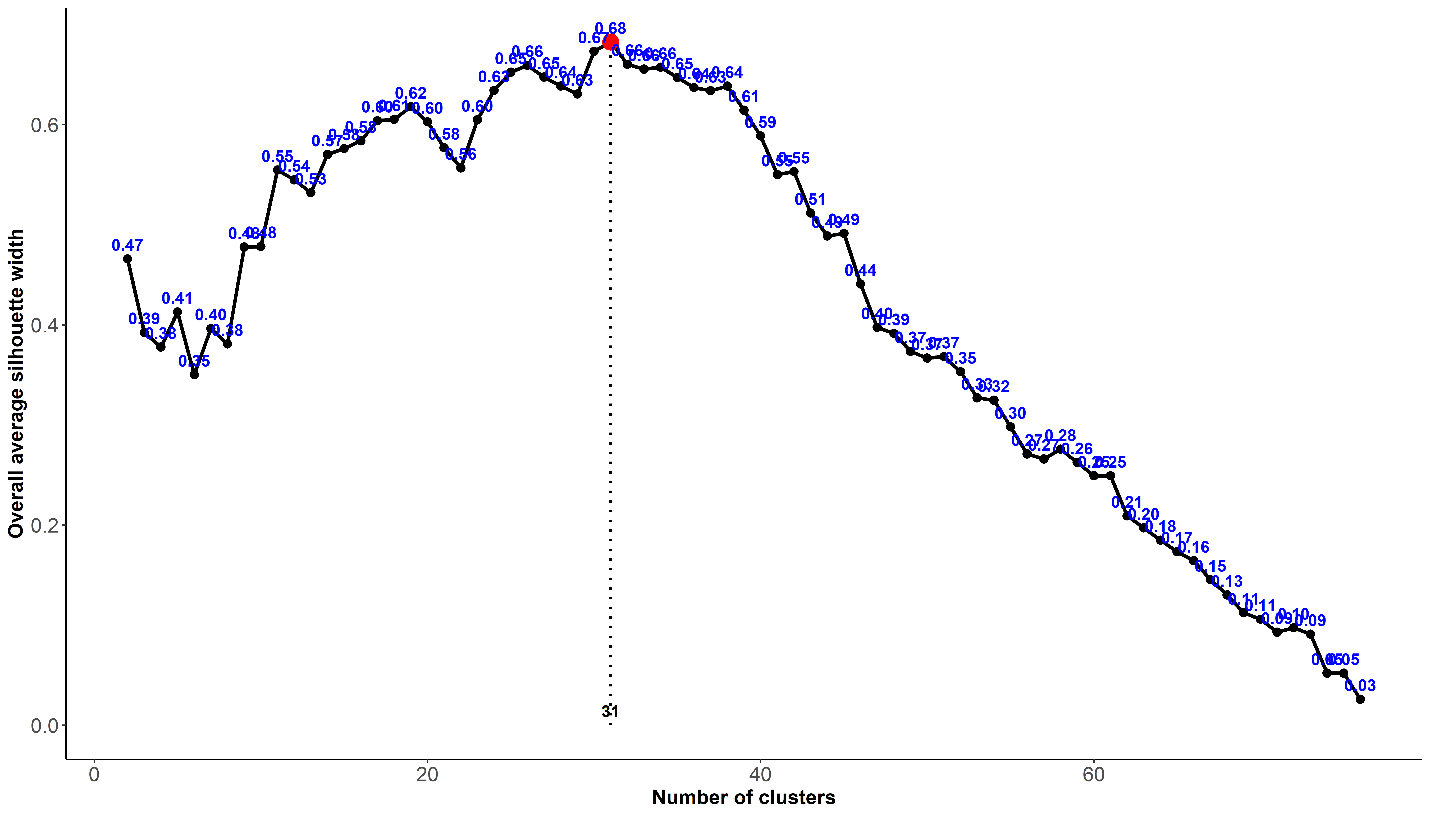


**Figure S17.** Profile plot for overall average silhouette width to detect the optimal partition for the comparisons in the network for chronic obstructive pulmonary disease.^15^ The x-axis refers to the investigated range of clusters (from two to $\sum_{i=1}^{39} \binom{\left| T_{i} \right|}{2}-1=77-1$, with $\left| T_{i} \right|$ being the number of treatments in the study $i$), and the y-axis refers to the overall average silhouette width for the corresponding investigated cluster. The red point indicates the optimal partition corresponding to the largest overall average silhouette width.

**
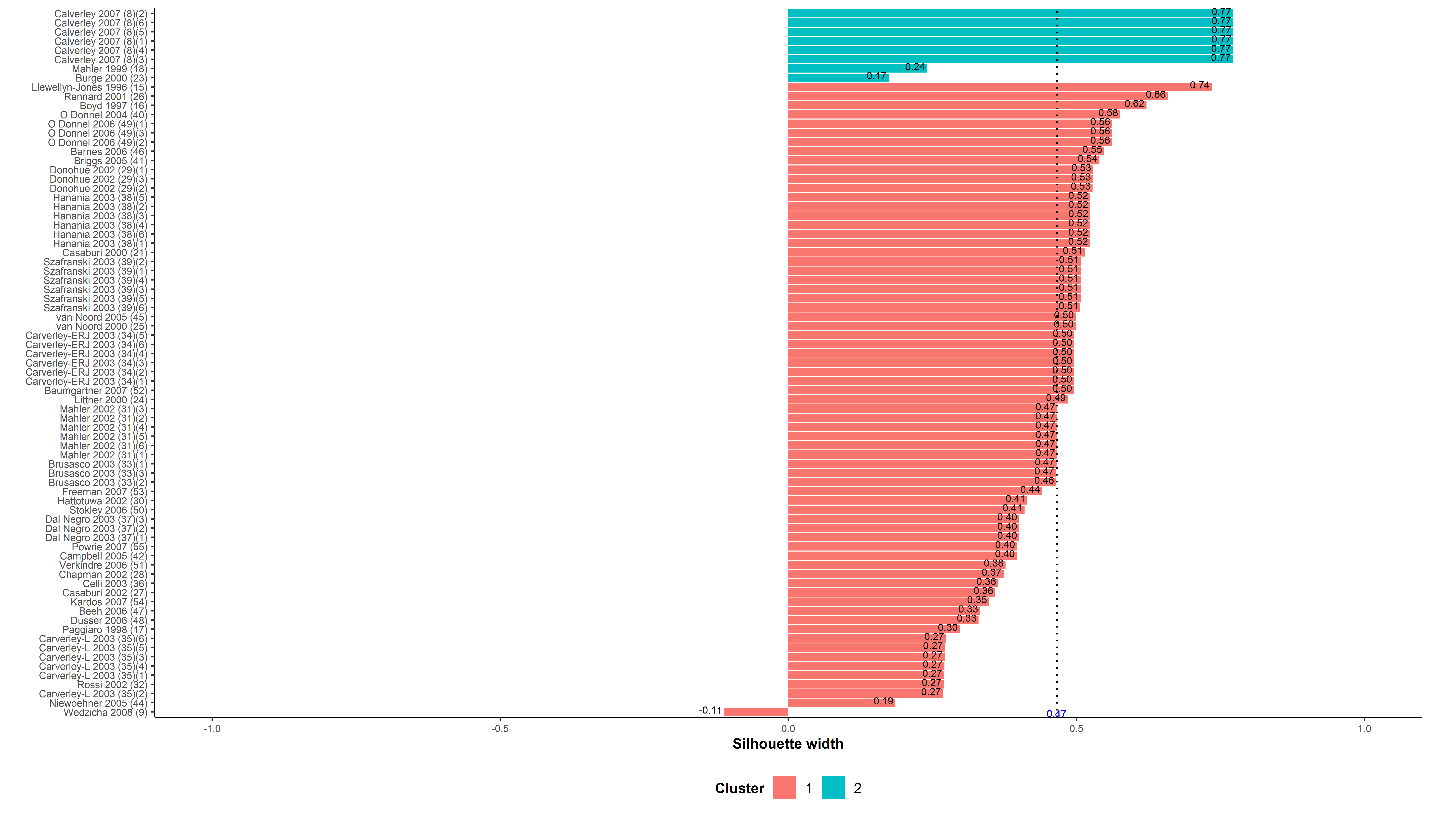
**

**Figure S18a.** Silhouette width plots for ***two clusters*** for the network on chronic obstructive pulmonary disease.^15^ Each bar refers to the silhouette width (x-axis) of the corresponding comparison (y-axis). The bars are sorted in decreasing order of the silhouette width within each cluster. The silhouette width value appears on each bar. Different colours refer to the different clusters. The vertical dotted line refers to the overall average silhouette width (number in blue).

**
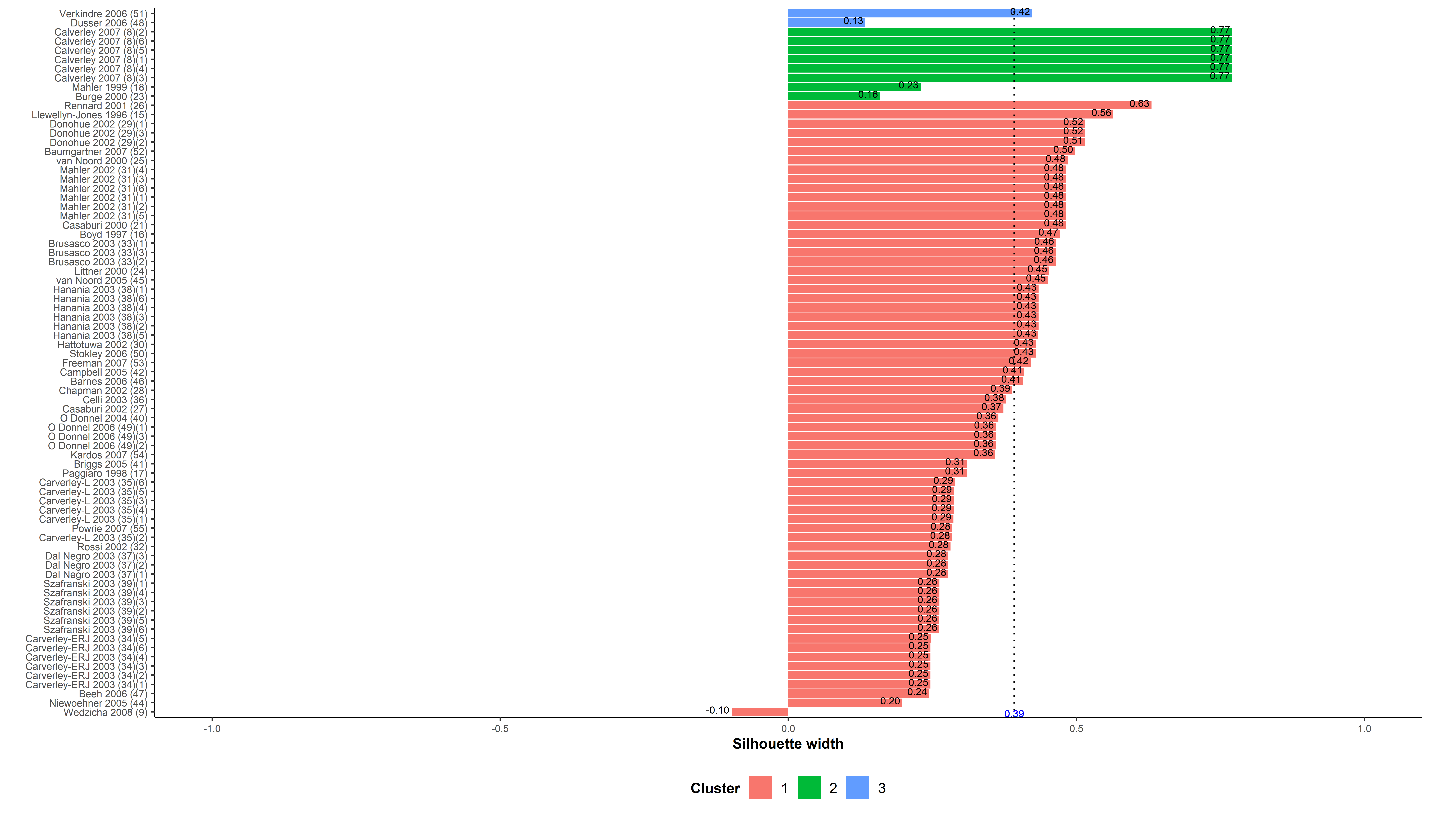
**

**Figure S18b.** Silhouette width plots for ***three clusters*** for the network on chronic obstructive pulmonary disease.^15^ Each bar refers to the silhouette width (x-axis) of the corresponding comparison (y-axis). The bars are sorted in decreasing order of the silhouette width within each cluster. The silhouette width value appears on each bar. Different colours refer to the different clusters. The vertical dotted line refers to the overall average silhouette width (number in blue).

**
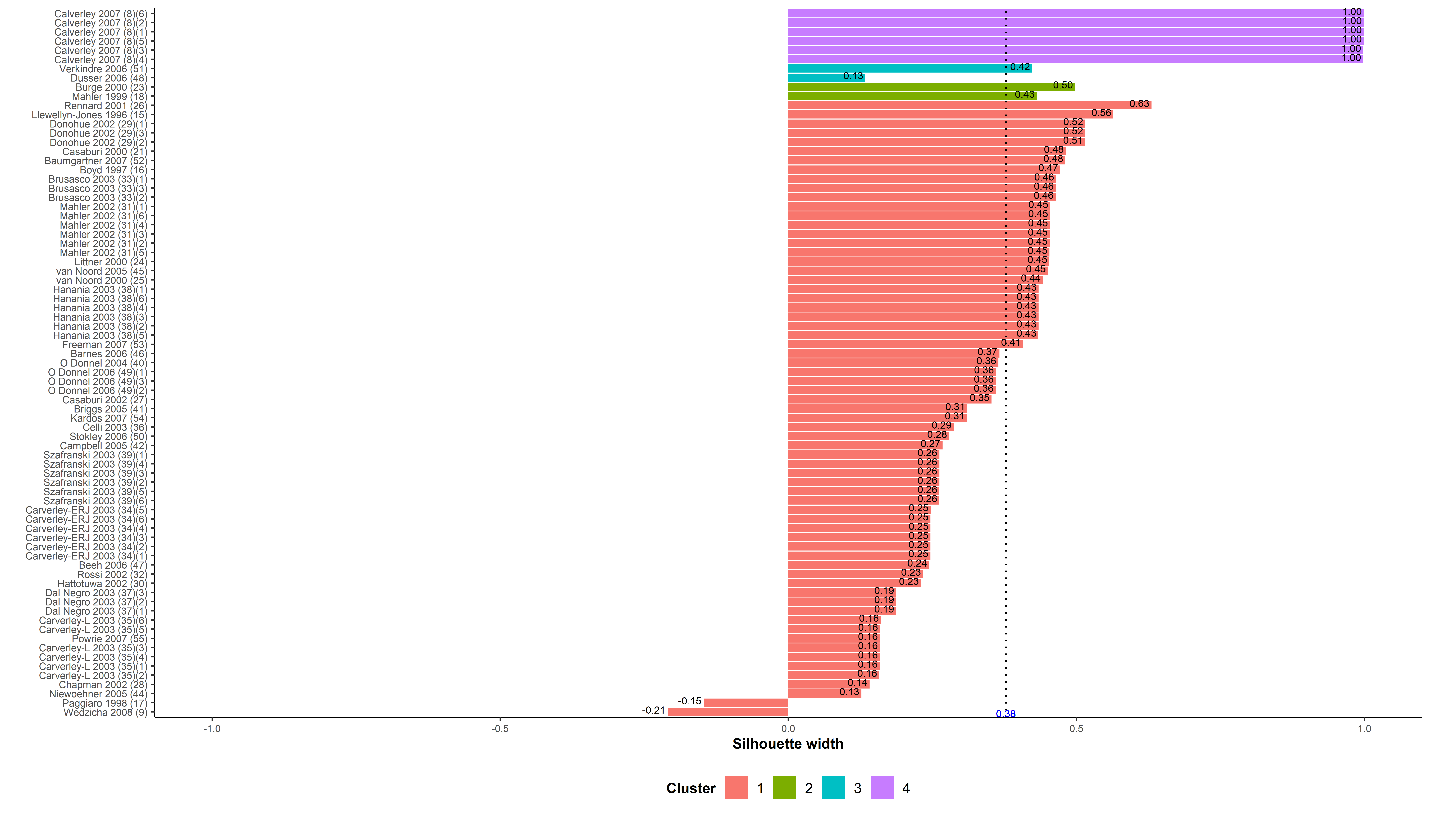
**

**Figure S18c.** Silhouette width plots for ***four clusters*** for the network on chronic obstructive pulmonary disease.^15^ Each bar refers to the silhouette width (x-axis) of the corresponding comparison (y-axis). The bars are sorted in decreasing order of the silhouette width within each cluster. The silhouette width value appears on each bar. Different colours refer to the different clusters. The vertical dotted line refers to the overall average silhouette width (number in blue).

**
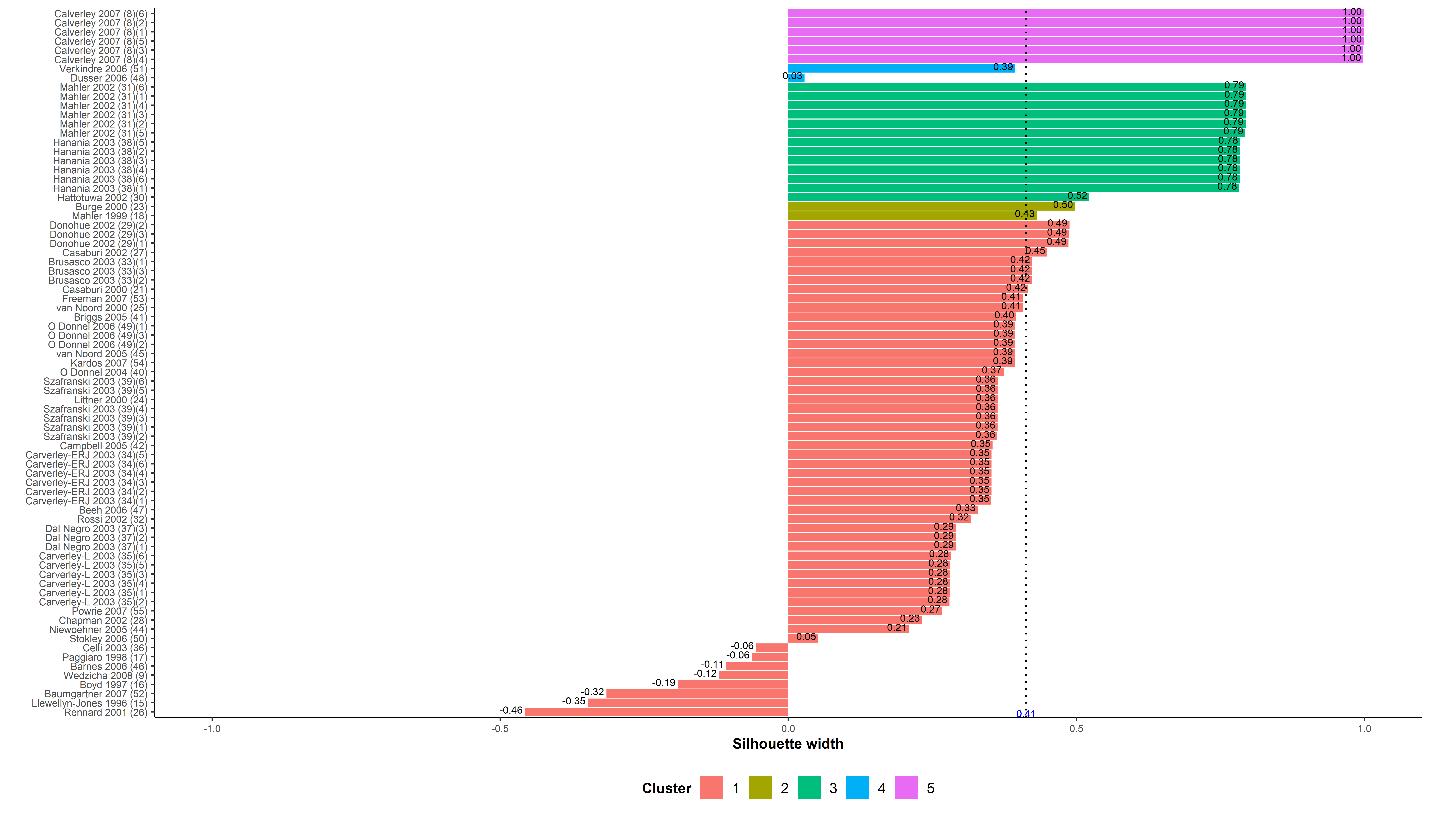
**

**Figure S18d.** Silhouette width plots for ***five clusters*** for the network on chronic obstructive pulmonary disease.^15^ Each bar refers to the silhouette width (x-axis) of the corresponding comparison (y-axis). The bars are sorted in decreasing order of the silhouette width within each cluster. The silhouette width value appears on each bar. Different colours refer to the different clusters. The vertical dotted line refers to the overall average silhouette width (number in blue).


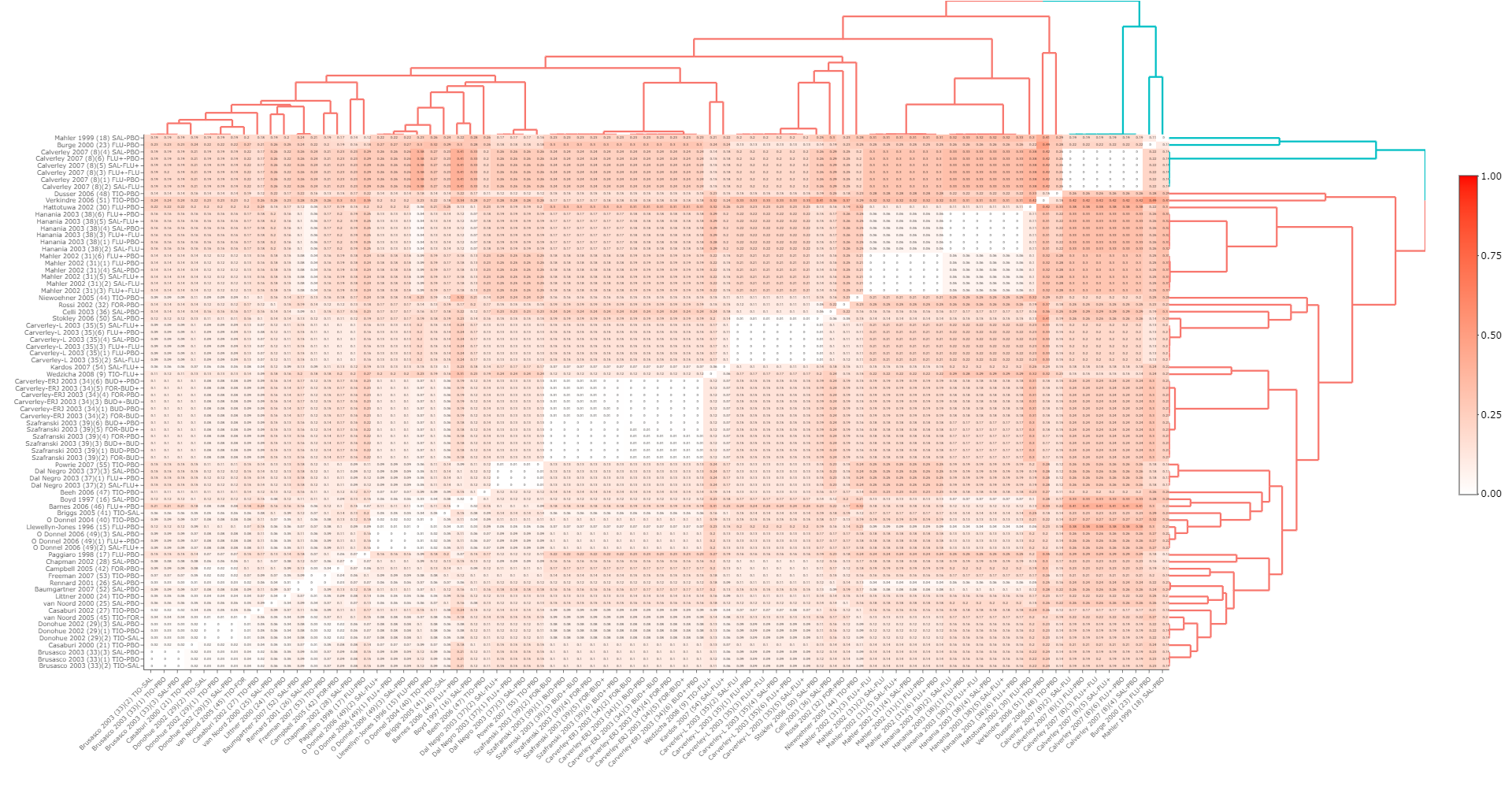
**Figure S19.** A heatmap on Gower's dissimilarity among the studies in the network and integrated dendrogram of two clusters. Darker shades of red refer to a higher dissimilarity between the compared studies. The leaves refer to the study name and corresponding treatment comparison. The network has multi-arm studies. The clusters are indicated with different colours of the branches. Analysis was performed on the network for chronic obstructive pulmonary disease.^15^

BUD, budesonide; BUD+, budesonide plus formoterol; FLU, fluticasone; FLU+, fluticasone plus salmeterol; FOR, formoterol; PBO, placebo; SAL, salmeterol; TIO, tiotropium.

**
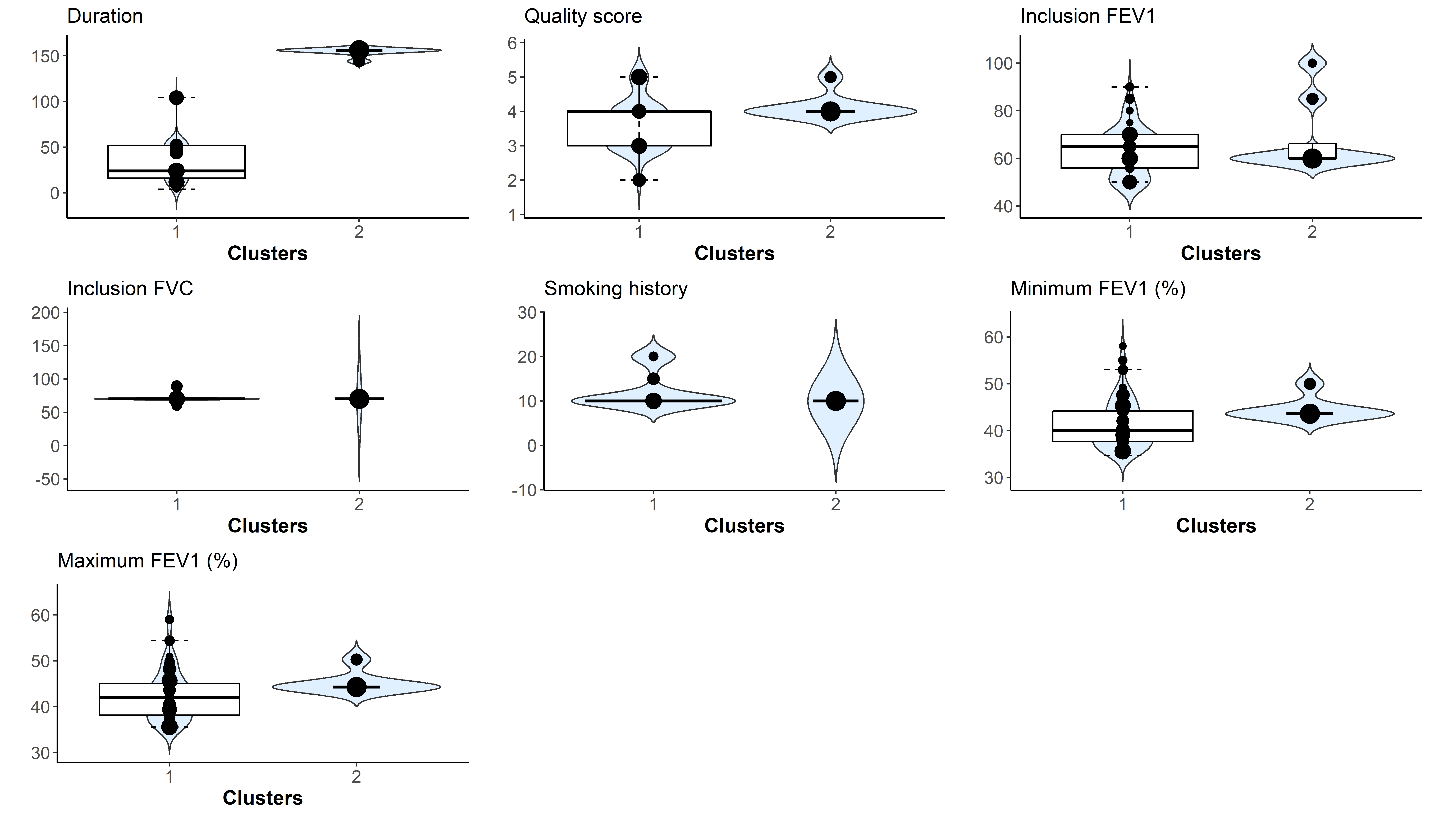
**

**Figure S20.** Violin plots (with integrated box plots and dots) for several quantitative characteristics extracted from the systematic review on chronic obstructive pulmonary disease.^15^ Each dot corresponds to a study in the corresponding cluster (x-axis), with the size of the dots being proportional to the total sample size of the studies: larger dots correspond to larger studies.

FEV1, forced expiratory volume in 1 second; FVC, forced vital capacity

**
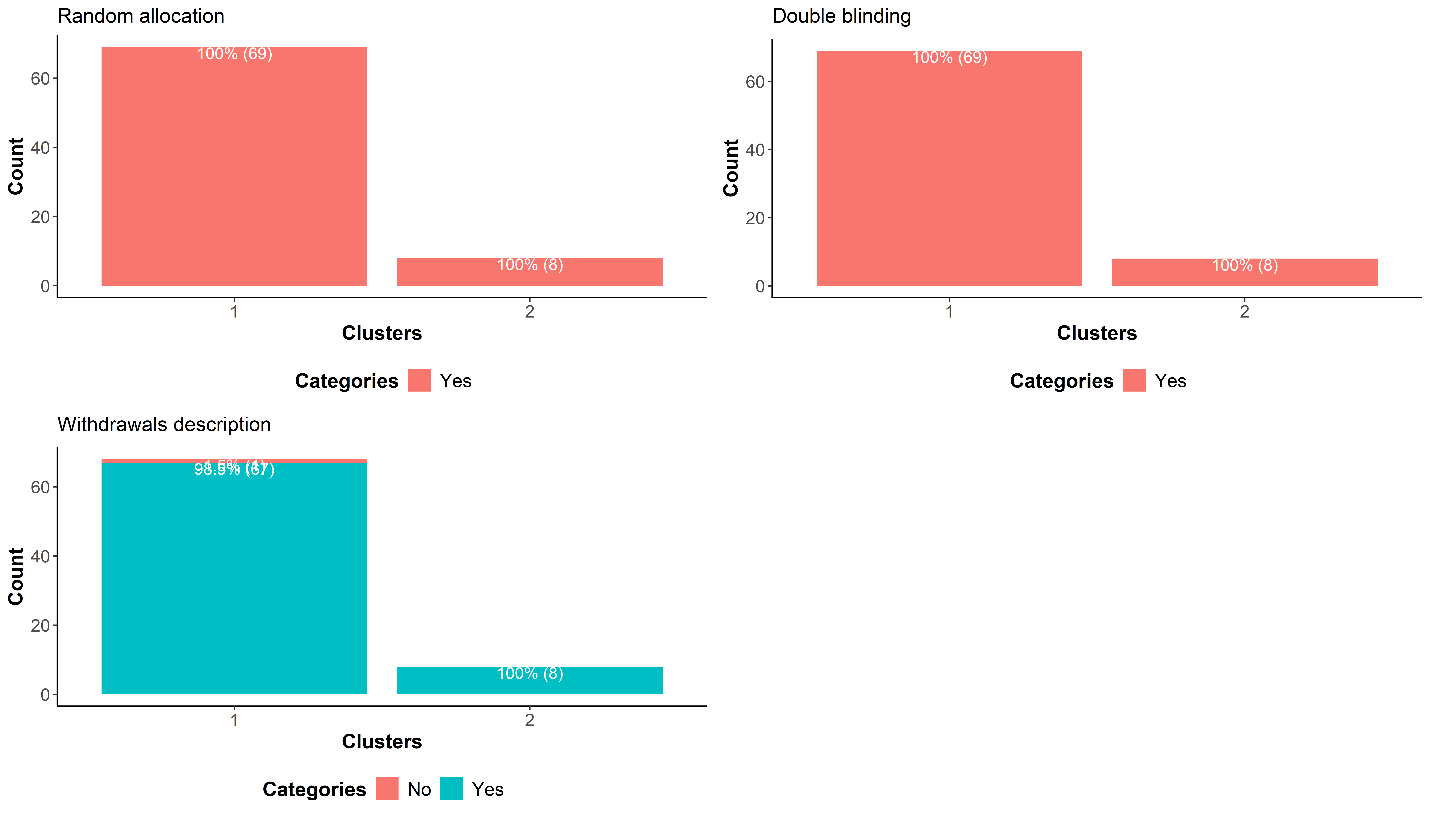
**

**Figure S21.** Stacked bar plots for several qualitative characteristics extracted from the systematic review on chronic obstructive pulmonary disease.^15^ The relative and absolute frequencies (in parenthesis) refer to each cluster on the x-axis.
