# Supplementary material for: Exploring the Transitivity Assumption in Network Meta‐Analysis: A Novel Approach and Its Implications
Source: Stat Med. 2025 Apr 10;44(7):e70068. doi: 10.1002/sim.70068 (PMC11983674; doi:10.1002/sim.70068)
Supplement: Supplementary file 2 — Supporting Information S2. [file SIM-44-0-s001.docx]

**Supporting Information for the *Methods* in the article 'Exploring the transitivity assumption in network meta-analysis: A novel approach and its implications'**

Loukia M. Spineli^1^  [Spineli.Loukia@mh-hannover.de](mailto:Spineli.Loukia@mh-hannover.de)

Katerina Papadimitropoulou^2^* [katerina.papadimitropoulou@amaris.com](mailto:katerina.papadimitropoulou@amaris.com)

Chrysostomos Kalyvas^3^* [chrysostomos.kal@gmail.com](mailto:chrysostomos.kal@gmail.com)

^1^Midwifery Research and Education Unit, Hannover Medical School, Hannover, Germany

^2^Health Economics and Market Access, Amaris Consulting, Lyon, France

^3^Biostatistics and Medical Informatics, Medical Faculty, University of Ljubljana, Vrazov trg 2, 1000 Ljubljana, Slovenia

*Contributed equally

**Supplementary Methods**

1. **Dealing with missing data**

Systematic reviews often encounter studies with at least one missing study or participant characteristic, which poses a challenge when investigating transitivity. Characteristics may not be reported for some comparisons because they are not believed to be effect modifiers for the compared treatments. In general, effect modifiers may vary across comparisons, so there is an argument for using different baseline characteristics for each comparison, leading to missing data for some characteristics. Deciding how to handle missing data is critical for ensuring the quality of the evaluation. The extent of missing data in each characteristic becomes a critical factor in determining whether to retain or exclude a characteristic from the dataset; for instance, it may be reasonable to exclude a characteristic with missing data in a large proportion of the studies.^1^ Retaining the characteristic may involve imputing the missing values in the respective studies or removing the studies with listwise deletion. Since the transitivity evaluation is aimed at the entire set of studies to determine whether synthesis is plausible, listwise deletion is counterproductive and inappropriate. Imputation requires the assumption that the mechanism behind missingness is random; otherwise, the quality of imputation would be compromised, questioning the credibility of the findings based on the completed dataset.

While methods exist for handling partially missing characteristics when conducting meta-regression (such as ^2^), there are currently no guidelines for addressing missing data in study characteristics within the transitivity evaluation. Since the present study ultimately aimed to investigate the (dis)similarity of comparisons based on the available evidence base and its limitations, we considered the following approach regarding the missing characteristics: we excluded from the dataset (i) characteristics completely missing in at least one non-single-study comparison, and (ii) characteristics with missing values in all but one studies for at least one (non-single-study) comparison. Excluding these missing characteristics by comparison rather than from the entire dataset would yield 'biased' within-comparison and across-comparison dissimilarities because they would be derived from different sets of characteristics.

**References**

1. Altman DG, Bland JM. Missing data. BMJ. 2007;334(7590):424.
2. Hemming K, Hutton JL, Maguire MG, Marson AG. Meta-regression with partial information on summary trial or patient characteristics. Stat Med. 2010;29(12):1312-24.
3. **Internal validity measure of clustering quality: Silhouette width**

The silhouette width proposed by Rousseeuw^1^ indicates how well-matched the studies are to their cluster compared to their neighbouring clusters. The index takes values in the range $\left[ -1,1 \right]$, with values closer to 1 indicating higher compactness and separation for the selected clustering partition, while values closer to -1 indicate possible misclassification.^1^ The silhouette width is calculated for each study as follows:

$$s\left( i \right)=\frac{b\left( i \right)-a\left( i \right)}{max\left\{ b\left( i \right),a\left( i \right) \right\}}=\left\{ \begin{aligned} 1-{a\left( i \right)}/{b\left( i \right) \text{if} a\left( i \right)<b\left( i \right)} \\ 0 \text{if} a\left( i \right)=b\left( i \right) \\ {b\left( i \right)}/{a\left( i \right)}-1 \text{if} a\left( i \right)>b\left( i \right) \end{aligned} \right.$$

with $b\left( i \right)$ being the minimum average distance between study $i$ and those in the other clusters, and $a\left( i \right)$ being the average distance between study $i$ and those found in the same cluster. For studies that comprise their own cluster, $s\left( i \right)=0$.^1^ Then the average of $s\left( i \right)$ yields the *average silhouette width* for the selected clustering partition $\mathcal{C}$:

$$S\left( \mathcal{C} \right)={\sum_{i=1}^{P} s\left( i \right)}/P$$

The index is accompanied by the silhouette width plot that illustrates $s\left( i \right)$ for all studies in their clusters in decreasing order alongside $S\left( \mathcal{C} \right)$. Studies with negative $s\left( i \right)$ or lower than $S\left( \mathcal{C} \right)$ indicate that the corresponding partitioning may not be optimal. The *overall* average silhouette width is also calculated for all studies.

The results of the silhouette width for a selected partitioning can be summarised in a plot called the *silhouette width plot*. The y-axis presents the studies sorted in decreasing order of their silhouette width (x-axis) and arranged in their cluster. The average silhouette width is indicated in each cluster alongside the overall average silhouette width at the plot's bottom. The overall average silhouette width is of particular interest as it aids in determining the optimal partitioning from a wide range of possible clusters (from 2 to $N-1)$. Then, the results are summarised in a *profile plot* that illustrates the behaviour of clustering across different partitions, with the y-axis referring to the overall average silhouette width calculated for each candidate cluster (x-axis). The partitioning corresponding to the higher overall average silhouette width is selected as the optimal one.

**References**

1. Rousseeuw PJ. Silhouettes: A graphical aid to the interpretation and validation of cluster analysis. J Comput Appl Math. 1987;20:53-65.

**3. List of (non-base) R packages for developing the *rnmamod* functions**

For the methods and figures:

1. stats ("as.dist", "cophenetic", "cutree", "hclust")
2. knitr
3. cluster
4. ggplot2
5. ggpubr
6. dendextend
7. heatmaply
8. scales

Citations of the listed R packages:

1. Core Team (2024). R: A Language and Environment for Statistical Computing. R Foundation for Statistical Computing, Vienna, Austria. <https://www.R-project.org/>
2. Xie Y (2024). knitr: A General-Purpose Package for Dynamic Report Generation in R. R package version 1.49, <https://yihui.org/knitr/>
3. Maechler, M., Rousseeuw, P., Struyf, A., Hubert, M., Hornik, K.(2023). cluster: Cluster Analysis Basics and Extensions. R package version 2.1.6.
4. H. Wickham. ggplot2: Elegant Graphics for Data Analysis. Springer-Verlag New York, 2016.
5. Kassambara A (2023). ggpubr: 'ggplot2' Based Publication Ready Plots. R package version 0.6.0, <https://CRAN.R-project.org/package=ggpubr>
6. Tal Galili (2015). dendextend: an R package for visualizing, adjusting, and comparing trees of hierarchical clustering. Bioinformatics. DOI: 10.1093/bioinformatics/btv428.
7. Galili, Tal, O'Callaghan, Alan, Sidi, Jonathan, Sievert, Carson (2017). “heatmaply: an R package for creating interactive cluster heatmaps for online publishing.” Bioinformatics. doi:10.1093/bioinformatics/btx657;. <https://doi.org/10.1093/bioinformatics/btx657>, <https://academic.oup.com/bioinformatics/article-pdf/doi/10.1093/bioinformatics/btx657/21358327/btx657.pdf>
8. Wickham H, Pedersen T, Seidel D (2023). scales: Scale Functions for Visualization. R package version 1.3.0, <https://CRAN.R-project.org/package=scales>
